# Diesel and Crude Oil Biodegradation by Cold-Adapted Microbial Communities in the Labrador Sea

**DOI:** 10.1128/AEM.00800-21

**Published:** 2021-09-28

**Authors:** Sean M. C. Murphy, María A. Bautista, Margaret A. Cramm, Casey R. J. Hubert

**Affiliations:** a Department of Biological Sciences, University of Calgary, Calgary, Alberta, Canada; University of Michigan—Ann Arbor

**Keywords:** Labrador Sea, subarctic, bioremediation, biostimulation, hydrocarbonoclastic bacteria, metagenomics, oil spill

## Abstract

Oil spills in the subarctic marine environment off the coast of Labrador, Canada, are increasingly likely due to potential oil production and increases in ship traffic in the region. To understand the microbiome response and how nutrient biostimulation promotes biodegradation of oil spills in this cold marine setting, marine sediment microcosms amended with diesel or crude oil were incubated at *in situ* temperature (4°C) for several weeks. Sequencing of 16S rRNA genes following these spill simulations revealed decreased microbial diversity and enrichment of putative hydrocarbonoclastic bacteria that differed depending on the petroleum product. Metagenomic sequencing revealed that the genus *Paraperlucidibaca* harbors previously unrecognized capabilities for alkane biodegradation, which were also observed in *Cycloclasticus*. Genomic and amplicon sequencing together suggest that *Oleispira* and *Thalassolituus* degraded alkanes from diesel, while *Zhongshania* and the novel PGZG01 lineage contributed to crude oil alkane biodegradation. Greater losses in PAHs from crude oil than from diesel were consistent with *Marinobacter*, *Pseudomonas_*D, and *Amphritea* genomes exhibiting aromatic hydrocarbon biodegradation potential. Biostimulation with nitrogen and phosphorus (4.67 mM NH_4_Cl and 1.47 mM KH_2_PO_4_) was effective at enhancing *n*-alkane and PAH degradation following low-concentration (0.1% [vol/vol]) diesel and crude oil amendments, while at higher concentrations (1% [vol/vol]) only *n*-alkanes in diesel were consumed, suggesting toxicity induced by compounds in unrefined crude oil. Biostimulation allowed for a more rapid shift in the microbial community in response to petroleum amendments, more than doubling the rates of CO_2_ increase during the first few weeks of incubation.

**IMPORTANCE** Increases in transportation of diesel and crude oil in the Labrador Sea will pose a significant threat to remote benthic and shoreline environments, where coastal communities and wildlife are particularly vulnerable to oil spill contaminants. Whereas marine microbiology has not been incorporated into environmental assessments in the Labrador Sea, there is a growing demand for microbial biodiversity evaluations given the pronounced impact of climate change in this region. Benthic microbial communities are important to consider given that a fraction of spilled oil typically sinks such that its biodegradation occurs at the seafloor, where novel taxa with previously unrecognized potential to degrade hydrocarbons were discovered in this work. Understanding how cold-adapted microbiomes catalyze hydrocarbon degradation at low *in situ* temperature is crucial in the Labrador Sea, which remains relatively cold throughout the year.

## INTRODUCTION

**FIG 1:**
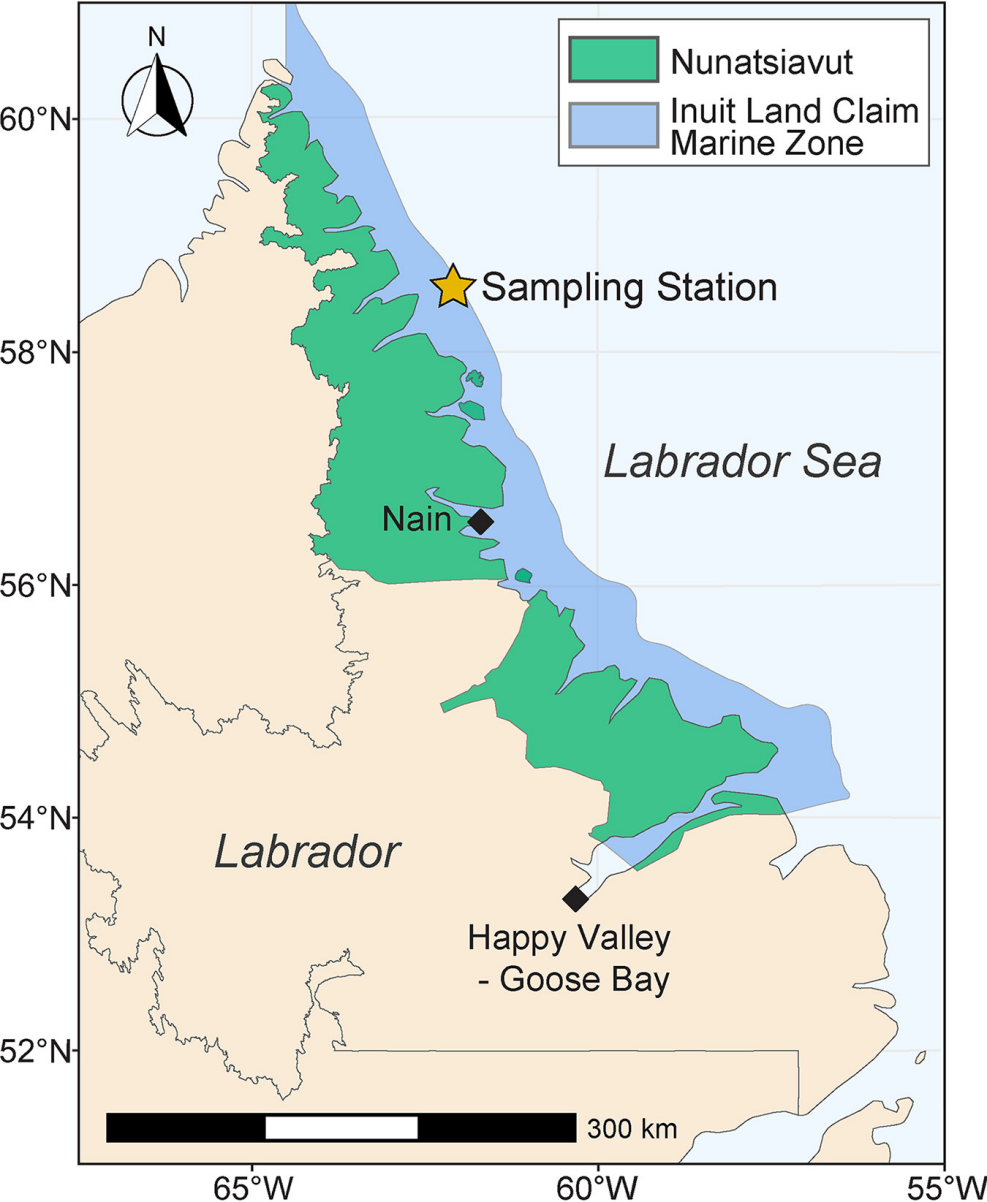
Map of Labrador and the Labrador Sea. Sampling station (star) marks where marine sediment was collected in triplicate in 2015 (cross-reference with Table S1 in the supplemental material). Sampling occurred within the marine zone of the Nunatsiavut Inuit Land Claim Agreement (blue shaded area along the coast).

The Labrador Sea (Fig. 1) faces heightened oil spill risk as shipping traffic in the region increases in response to longer ice-free seasons within connected Arctic corridors (1–3). In addition, oil production offshore Labrador is expected to increase with the government of Newfoundland and Labrador planning to double its extraction of the resource by the year 2030. Oil spill research has expanded rapidly over the past decade, better equipping jurisdictions to manage future spills (4). The behavior and fate of spilled oil in the Labrador Sea, however, remains difficult to predict on account of accessibility to the area, cold ocean temperature, and prolific sea ice. A better understanding of oil spill dynamics in this region and other subarctic areas should help in managing future oil releases in these vulnerable environments.

Oil contamination leaves a lasting impact on northern ecosystems already facing cumulative negative effects from climate change, pollution, and overexploitation of resources (5, 6). Despite the heavy occurrence of icebergs, first year sea ice, and an increasing occurrence of mobile Arctic multiyear sea ice (7) in the Labrador Sea, current environmental assessment reports for the region highlight a “limited knowledge base” regarding oil spills in ice impacted waters (8). Concerns have been expressed by Labradoreans over the impact that an oil spill may have on coastal areas (8) given bioaccumulation of petroleum constituents within fish and other marine wildlife that local communities rely on for food (5). Labrador’s remoteness and distance from oil spill response resources located in more southern cities and ports (i.e., St. John’s) make local wildlife and coastal communities inhabited by Nunatsiavut Inuit, the Southern Inuit of NunatuKavut, and Sheshatshiu and Mushuau Innu, more vulnerable to oil spill contaminants. The 1989 Exxon Valdez oil spill underscored the significant risks to shoreline ecosystems posed by transporting crude oil via ocean shipping (9). In addition, the Canadian Coast Guard has voiced concern over spills of diesel because of its heavy use as the fuel for maritime transportation in the Arctic, because of its eventual replacement of bunker fuel in larger cargo ships, and because of the high volumes of diesel shipped by sea for electricity generation in northern communities (10).

With increasing oil spill risk there is a growing need for reliable marine bioindicators of environmental impact (11, 12) and effective strategies for dealing with hydrocarbon contaminants in the environment (13–16). Microbial responses to hydrocarbons have been studied thoroughly (17) with over 330 genera of *Bacteria* and *Archaea* identified with metabolisms, degradation pathways, and genes involved in hydrocarbon oxidation (18). Hydrocarbonoclastic bacteria (HCB), a large proportion of which are members of the class *Gammaproteobacteria*, can use multiple carbon sources but display a preference for hydrocarbons (19). Some HCB, mostly marine bacteria, have been described as obligate, i.e., they use hydrocarbons almost exclusively (20). *Alcanivorax* and *Cycloclasticus* are commonly detected in oil spill studies and are typically implicated in the biodegradation of alkane and cyclic hydrocarbons, respectively (21). Other groups often considered obligate, such as *Oleiphilus*, *Oleispira*, and *Thalassolituus*, are also commonly enriched in the marine environment following oil spills (19–21). Despite being present in low abundance initially, accidental releases of oil provoke rapid proliferation of these and other hydrocarbon-degrading taxa (22, 23).

Low temperatures affect rates of oil biodegradation by reducing the solubility of certain oil compounds (24). This is especially important given that sea surface temperatures along the Labrador Shelf remain relatively cold throughout the year, ranging from –2°C to 10°C (8). Microbial communities capable of responding to hydrocarbons at low temperature can differ from those that respond at higher temperatures (25–27). HCB responses also depend on the concentration of hydrocarbons released to the environment (28) and the composition of the petroleum product, i.e., its proportion of alkanes (29) and aromatic hydrocarbons (24, 30). The rate and extent of oil degradation can also be limited by the low abundance of nitrogen (N) and phosphorus (P) in marine environments (31, 32). While spilled oil provides a rich carbon and energy source for HCB, it contains minimal available N and P for meeting the requirements that all microorganisms share for synthesizing nucleic acids and proteins. For every gram of hydrocarbon consumed, microorganisms require ∼150 mg N and ∼30 mg P (33, 34). Adding N and P following an oil spill allows indigenous microbial populations to overcome these environmental limitations. This biostimulation strategy was successfully employed in response to the Exxon Valdez oil spill, using the fertilizer Inipol EAP 22 (35).

In an open ocean context, sinking oil constitutes a considerable fraction of major spills, i.e., 25% during Ixtoc I (36) and an estimated 15% during Deepwater Horizon (11, 37). Partitioning of hydrocarbons between sediments and adjacent food webs represents an important exposure route (38) leading to disease, impaired health, and mortality in a wide range of organisms (39). Hydrocarbon biodegradation by benthic microbial communities is an important consideration when assessing the fate of oil in areas where shipping traffic and oil production are expected to increase. This is the first study to uncover how cold-adapted microbial communities in the Labrador Sea respond to hydrocarbon inputs of diesel or crude oil, as well as nutrient stimulation.

## RESULTS

### Biostimulation for diesel fuel and crude oil biodegradation.

**FIG 2:**
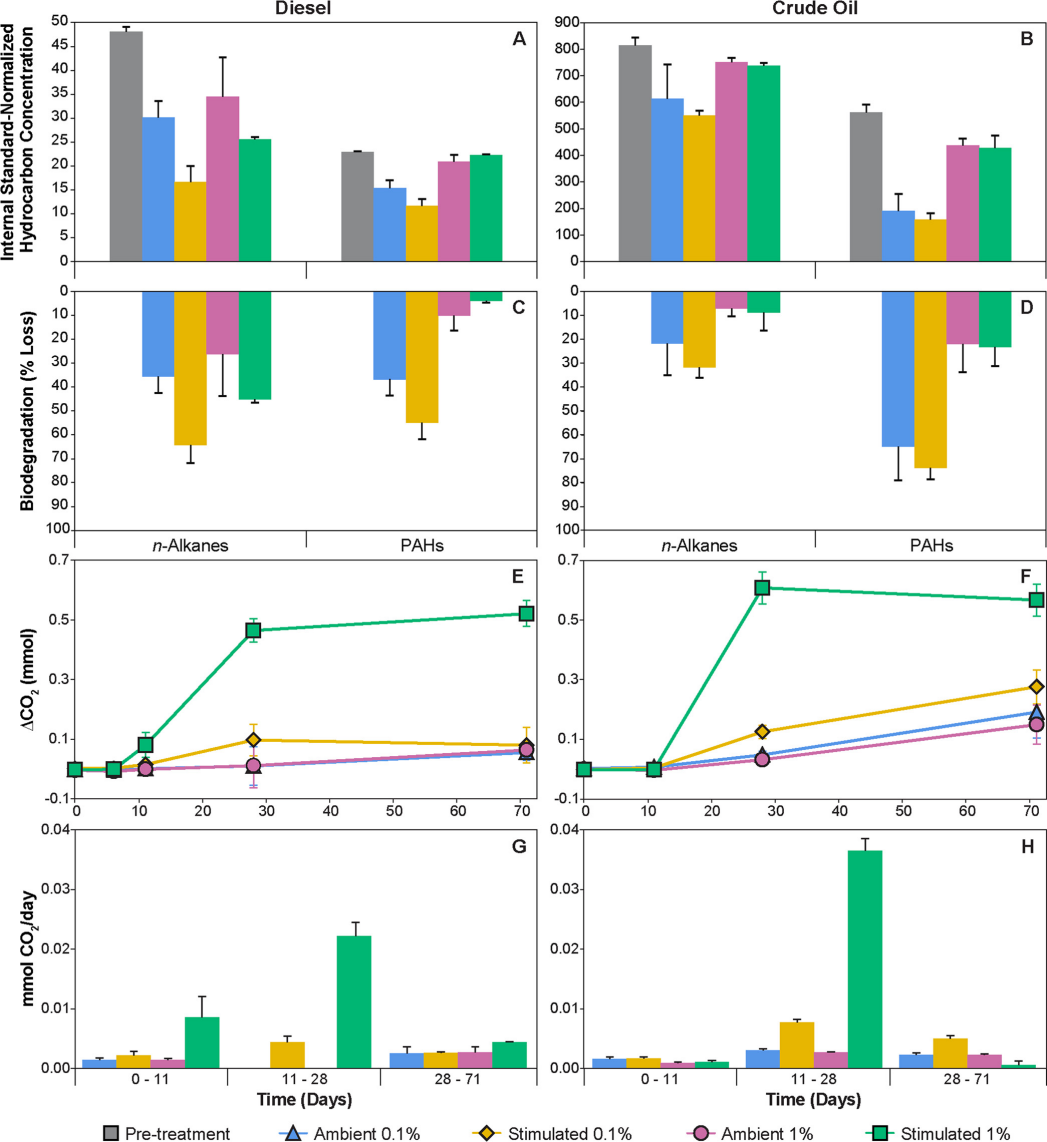
Hydrocarbon biodegradation within 0.1 and 1% (vol/vol) diesel (A, C, E, and G)- and crude oil (B, D, F, and H)-amended microcosms with nutrient stimulation or without (ambient). (A and B) Concentration of hydrocarbon compound groups at day 0 (pre-treatment) and after 71 days of incubation at 4°C. Hydrocarbon concentrations in diesel were obtained by normalizing compound peak areas against 1,3+3,9+2,10+3,10-dimethylphenanthrene, and in crude oil by normalizing against 17α(H),21β(H)-hopane. (C and D) Biodegradation conveyed as losses of measured *n*-alkanes (C_12_-C_25_) and PAHs from pre-treatment levels. (E to H) CO_2_ production (E and F) determined by GC is plotted as the difference between hydrocarbon-amended and -unamended microcosms and was used to estimate rates of CO_2_ production associated with hydrocarbon biodegradation (G and H) (CO_2_ concentrations for unamended controls are shown in Fig. S1). Error bars represent standard errors of triplicate microcosms of the same treatment.

Nutrient addition enhanced low temperature (4°C) biodegradation of *n*-alkanes (C_12_-C_25_) in all diesel- or crude oil-amended microcosms but had the least impact on 1% crude oil, as revealed by gas chromatography-mass spectrometry (GC-MS) (Fig. 2A to D). This effect is evident in the ratio of *n*-C_17_/pristane decreasing in all diesel amendments, but only in the 0.1% crude oil amendments (see Table S2). Alkanes were significantly more degraded in diesel (43% loss) than crude oil (17% loss) (F_1,21_ = 11.97, *P* = 0.002), despite comprising a similar initial proportion of the total measured hydrocarbons in both cases (Fig. 2A and B). Biostimulation also promoted biodegradation of polycyclic aromatic hydrocarbons (PAHs) in the presence of low (0.1%) but not high (1%) concentrations of diesel or crude oil (Fig. 2A to D), as reflected by 2MN/1MN ratios (see Table S2 in the supplemental material). PAHs were significantly more biodegraded in 0.1% crude oil (70%) than 0.1% diesel (46%) (F_1,9_ = 8.96, *P* = 0.02).

Effects of hydrocarbon biodegradation stimulated by nutrient addition were also monitored by assessing headspace O_2_ consumption and CO_2_ production (Fig. 2E and F; see also Fig. S1 in the supplemental material). Biostimulation resulted in significantly more CO_2_ generation among 1% diesel and crude oil amendments incubated at 4°C. Nutrient stimulation further increased rates of CO_2_ generation, which reached maximum levels between 11 and 28 days in both 0.1 and 1% amendments. No significant changes in CO_2_ were observed in any sediment-free or killed control microcosms (see Fig. S1E, F, I, and J). The combination of nutrient stimulation with 1% diesel led to complete oxygen consumption after 28 days (see Fig. S1E), resulting in anoxic conditions impacting subsequent CO_2_ generation in these microcosms. There was no difference in CO_2_ production between 0.1 and 1% amendments in the presence of ambient levels of nutrients, suggesting that seawater concentrations of N and P are limiting and require replenishment in order for microbial communities to contend with larger petroleum inputs. Ammonium-N persisted in the ambient nutrient control microcosms that were not amended with diesel or crude oil and in all nutrient-stimulated treatments whether amended with petroleum or not (see Fig. S2).

### Microbial community changes in response to hydrocarbon input and biostimulation.

**FIG 3:**
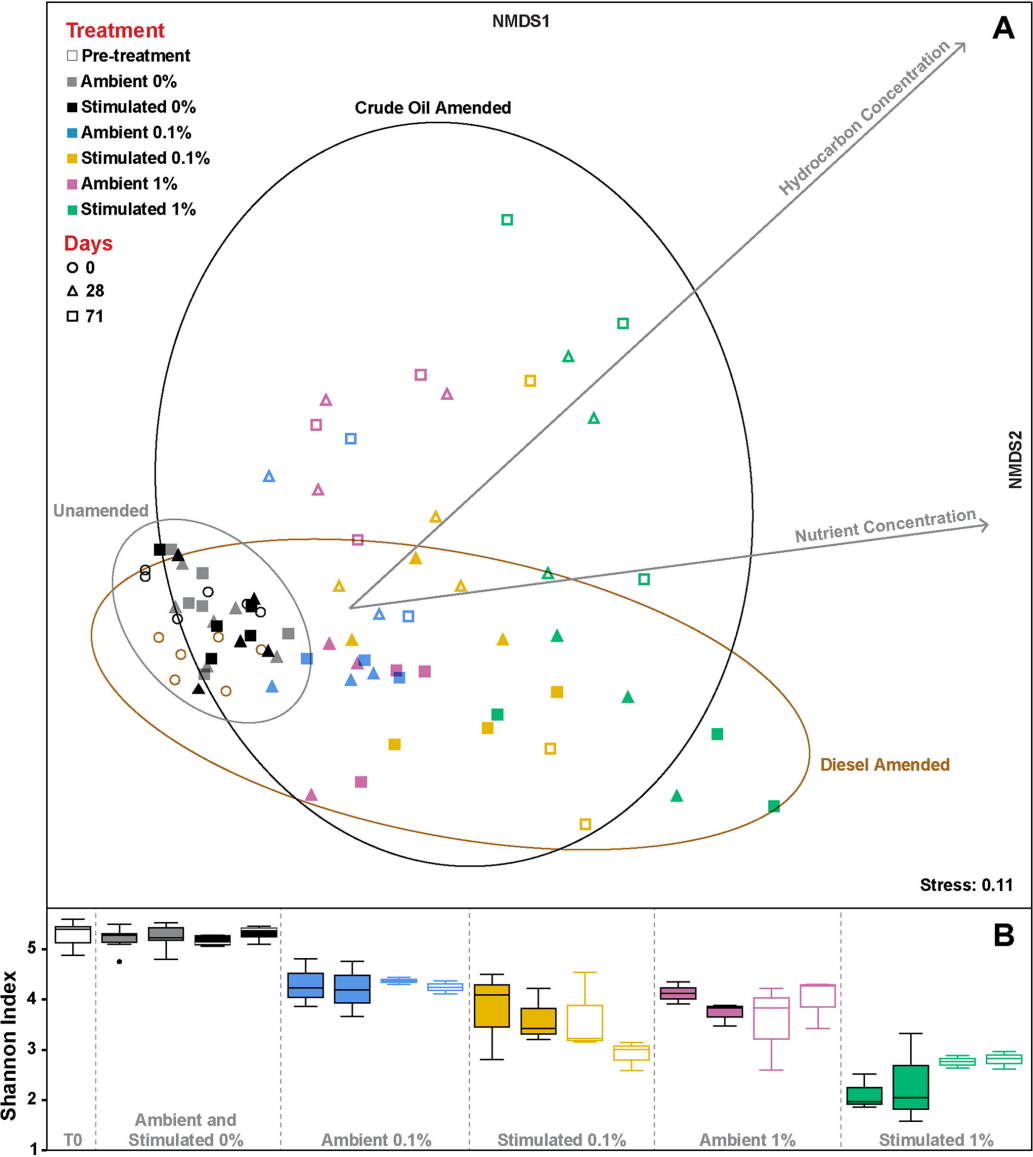
(A) Microbial community structure similarity among unamended (black and gray points), diesel-amended (solid-color points) and crude oil-amended (unfilled colored points) Labrador sediment microcosms incubated at 4°C for 71 days visualized by nonmetric multidimensional scaling (NMDS) of Bray-Curtis values for each community using 0.95 confidence cutoffs (ellipses). The lengths of the two factor arrows indicate the contribution of hydrocarbon and nutrient concentration variables to the dissimilarity between treatment group microbial communities. Significant fit and strong correlation were observed with hydrocarbon concentration (*r*^2^ = 0.45, *P* = 0.001), nutrient concentration (*r*^2^ = 0.34, *P* = 0.001), and type of petroleum product (*r*^2^ = 0.57, *P* = 0.001). The stress value corresponds to disagreement between the two-dimensional configuration and predicted values, with stress <0.2 considered a good representation. Triplicates are plotted for all conditions except for crude oil treatment “Ambient 0.1%,” for which one replicate was lost. Twelve microcosms were assessed to obtain day 0 (pre-treatment) microbial community data. Associated ANOSIM statistics and simper results are presented in Data Set S2. (B) Shannon Index (i.e., α-diversity) for each treatment group, with the central band representing the median among replicates, the box containing 50% of observations, and whiskers extending to the lowest and highest values excluding outliers (separate points). Aside from the pre-treatment box all treatments have two pairs, the first being the day 28 results, and the second being that of day 71.

Microbial community structure during 71 days of 4°C incubation was heavily influenced by both the type and concentration of petroleum product and whether nutrients were added in the presence of hydrocarbons (Fig. 3A). Nonmetric multidimensional scaling (NMDS) of Bray-Curtis dissimilarity revealed unincubated (day 0) and all unamended communities (28 and 71 days, regardless of nutrient stimulation) cluster together, signifying little community change in the absence of hydrocarbons (Fig. 3A, “unamended” ellipse; R-stat = 0.3, *P* = 0.001). The introduction of diesel or crude oil significantly influenced community structure (*r*^2^ = 0.57, *P* = 0.001), resulting in clusters according to diesel or crude oil amendment that were significantly dissimilar from each other (R-stat = 0.667, *P* = 0.001) and from the community structure in unamended microcosms (Fig. 3A; see also Data Set S3). Higher concentrations of diesel or crude oil were significantly correlated with changes in community structure (*r*^2^ = 0.45, *P* = 0.001) with significant dissimilarity between communities in 0.1 and 1% amended microcosms, particularly within the crude oil cluster (R-stat = 0.52, *P* = 0.001) more than the diesel cluster (R-stat = 0.25 *P* = 0.001). Nutrient stimulation also influenced these communities (*r*^2^ = 0.34, *P* = 0.001), which were significantly dissimilar between ambient and nutrient stimulated treatments within the diesel cluster (R-stat = 0.39, *P* = 0.001) more than the crude oil cluster (R-stat = 0.1468, *P* = 0.026).

Microbial α-diversity was greatest prior to incubation or amendment with petroleum products, as revealed by the Shannon Index (Fig. 3B) and the number of operational taxonomic units (OTUs) (see Fig. S3). No significant changes in either of these parameters were observed throughout 71 days of 4°C incubation in the unamended controls, whereas diversity always dropped in the presence of diesel or crude oil regardless of their concentrations or nutrient biostimulation. Microbial community structure (Fig. 3A) and α-diversity (Fig. 3B) exhibited the largest changes within the first 28 days of incubation, concomitant with the highest rates of CO_2_ generation (Fig. 2E and F). Microbial communities in diesel-amended microcosms shifted more within the first 28 days of incubation (R-stat = 0.3, *P* = 0.001) and experienced the greatest declines in Shannon Index (Fig. 3B) and OTU richness (see Fig. S3), compared to the 28- to 71-day period (R-stat = 0.07, *P* = 0.09), suggesting little change in the microbial community during the later interval. While hydrocarbon-amended and nutrient-stimulated microcosms exhibited the greatest declines in diversity, nutrient stimulation also resulted in the highest yields in DNA concentration (see Fig. S4), consistent with low temperature growth of a limited number of taxa in the presence of hydrocarbons.

### Microbial response to hydrocarbon input and biostimulation at 4°C.

**FIG 4:**
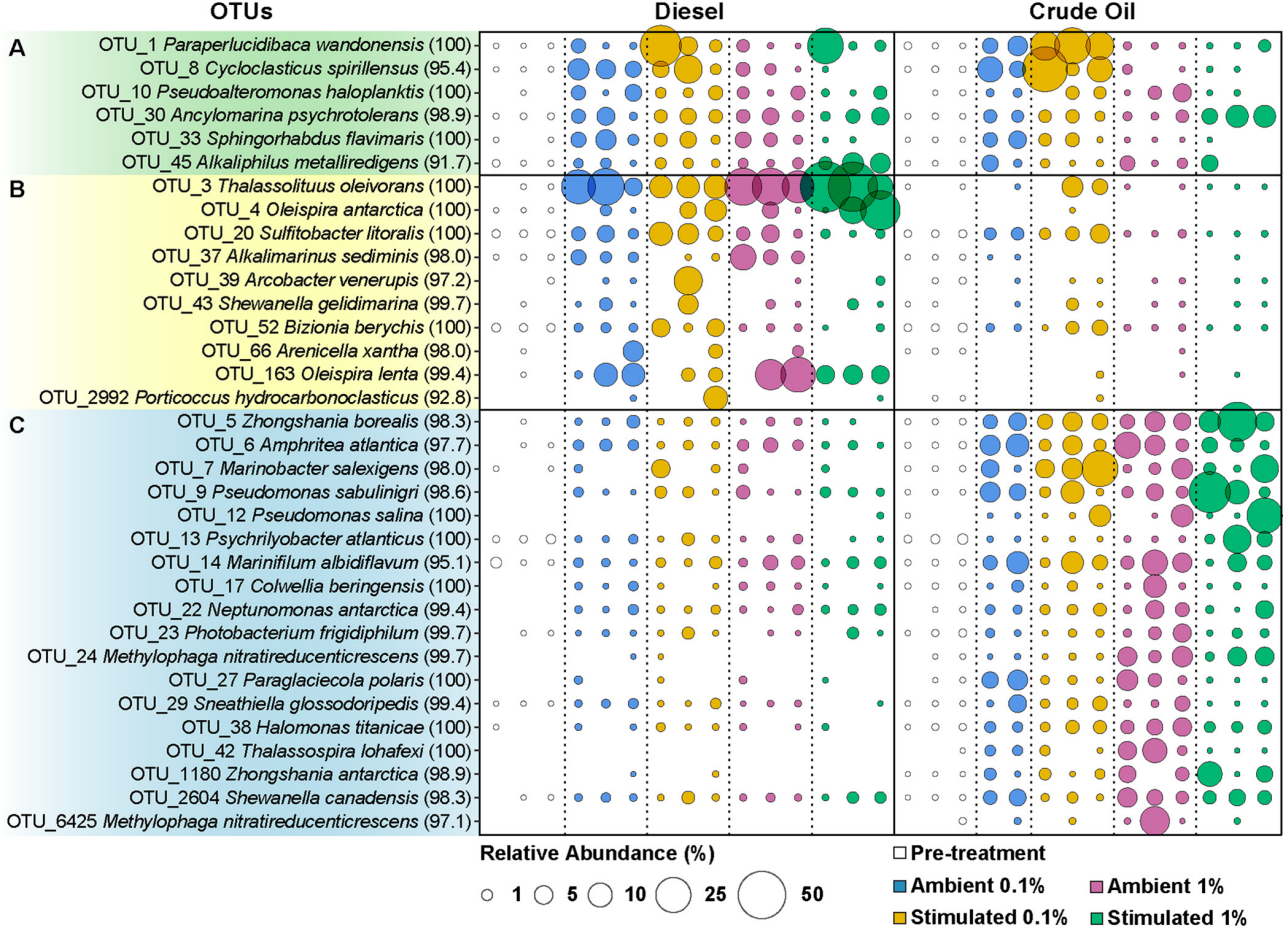
Relative sequence abundance of OTUs with significant associations to diesel or crude oil input after 71 days of incubation at 4°C. Bubble size indicates OTU relative abundance (%), with missing bubbles indicating no sequence reads. OTUs are labeled according to closest cultured relatives identified following NCBI BLAST searching with sequence identities (%) shown in parentheses. OTUs are grouped according to significant associations identified by “indicspecies” as those strongly correlated with both diesel and crude oil contamination (A), those correlated with the presence of diesel only (B), or crude oil only (C).

Microbial groups affiliated to known aliphatic and aromatic hydrocarbon degrading taxa, originally at low levels at day 0, became abundant after 28 and 71 days of 4°C incubation in the presence of diesel or crude oil. Among 56 OTUs found to contribute to at least 50% of the dissimilarity between all groups tested using analysis of similarities (ANOSIM) (see Fig. S5 and Data Set S3), 34 were significantly associated with diesel and/or crude oil amendments (Fig. 4; see also Data Set S4). Of these OTUs, six were significantly associated with both diesel and crude oil amendments. Included in this group were *Paraperlucidibaca* OTU_1 and *Cycloclasticus* OTU_8, both exhibiting increases in abundance mainly in the 0.1% amendments. Diesel was significantly correlated with 10 OTUs, including *Oleispira* OTUs 4 and 163 and *Thalassolituus* OTU_3 that exhibited the greatest increases in relative abundance. Crude oil amendment led to 18 OTUs that were selectively enriched, with *Zhongshania* OTU_5, *Marinobacter* OTU_7, and Pseudomonas OTUs 9 and 12 exhibiting the greatest increases in relative abundance.

**FIG 5:**
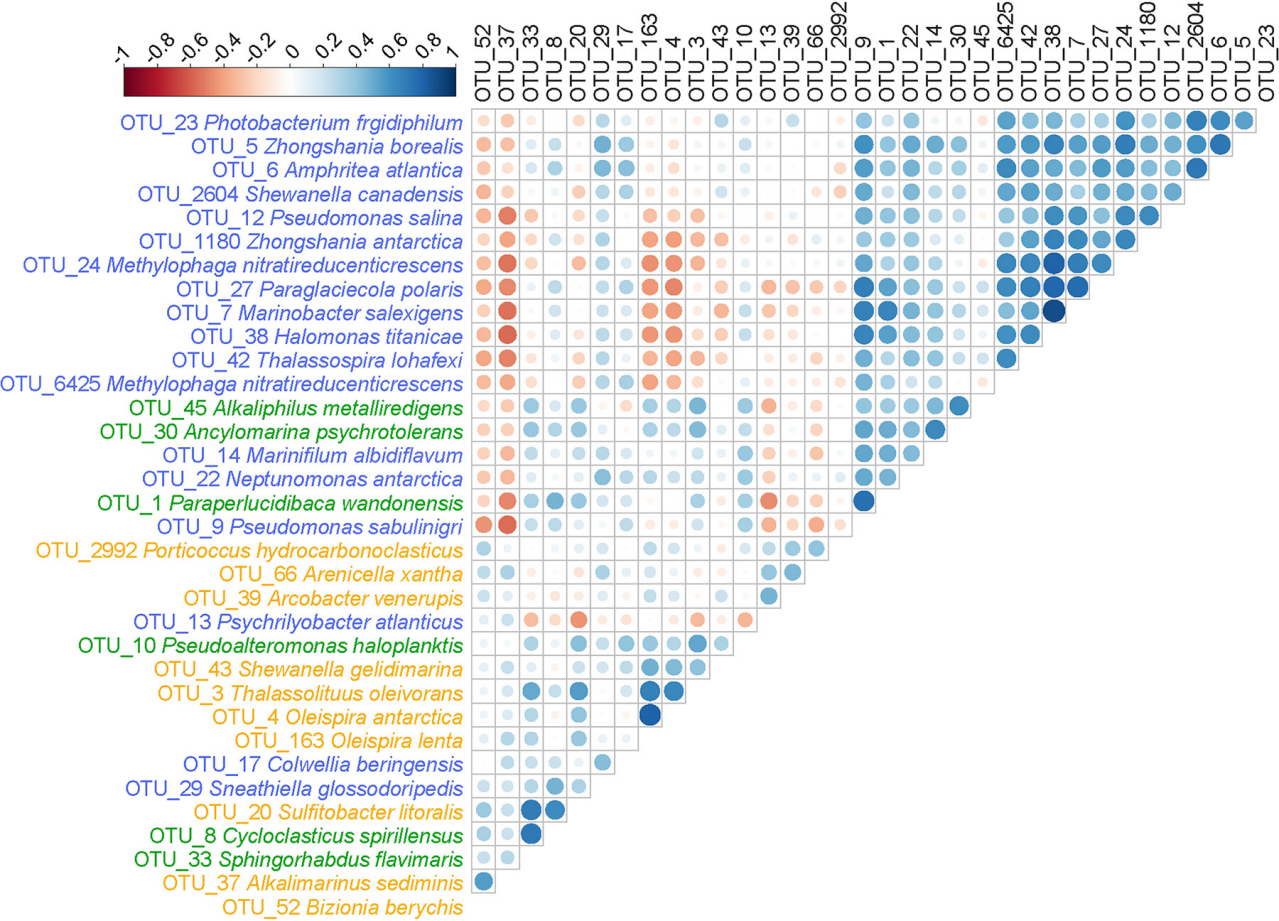
Spearman correlation matrix highlighting positive (blue) or negative (red) cooccurrence relationships between hydrocarbon associated OTUs (cross-reference with Data Set S4). Analysis utilized rarefied sequences from all incubations, both amended and unamended, at 0, 28, and 71 days of incubation. Empty boxes indicate no correlation was found between pairs, while the larger the circle size and darker the color, the stronger the correlation between associated OTUs. The highlighting of the OTUs indicates they were significant indicators of crude oil (blue), diesel (yellow), and both crude oil and diesel (green) enrichment.

Specific bacteria were found to be significantly associated with specific diesel or crude oil concentrations or nutrient biostimulation (Fig. 4; see also Data Set S4), in some cases exhibiting strong cooccurrence with other OTUs (Fig. 5). In diesel-amended biostimulation treatments, *Oleispira* OTU_4 was significantly associated with 1% diesel, whereas *Porticoccus* OTU_2992 was prevalent only in 0.1% diesel, suggesting a differential affinity or tolerance for diesel between different taxa. Interestingly, the presence of *Cycloclasticus* OTU_8 was significant in all 0.1% diesel and crude oil amendments, suggesting broad affinity by this group but only at lower concentrations. Within crude oil amendments, *Marinobacter* OTU_7 was significantly associated with nutrient biostimulation, whereas *Colwellia* OTU_17 was only prevalent at the lower ambient nutrient condition, suggesting differential responses by putative oil-degrading bacteria to N and P addition. Diesel-associated *Oleispira* OTUs (4 and 163) shared a strong positive correlation with each other and with *Thalassolituus* OTU_3 (Fig. 5). These three diesel-associated OTUs exhibited strong negative correlations with many of the crude oil-associated OTUs, such as *Zhongshania* OTUs 5 and 1180, *Marinobacter* OTU_7, and Pseudomonas OTU_12.

**FIG 6:**
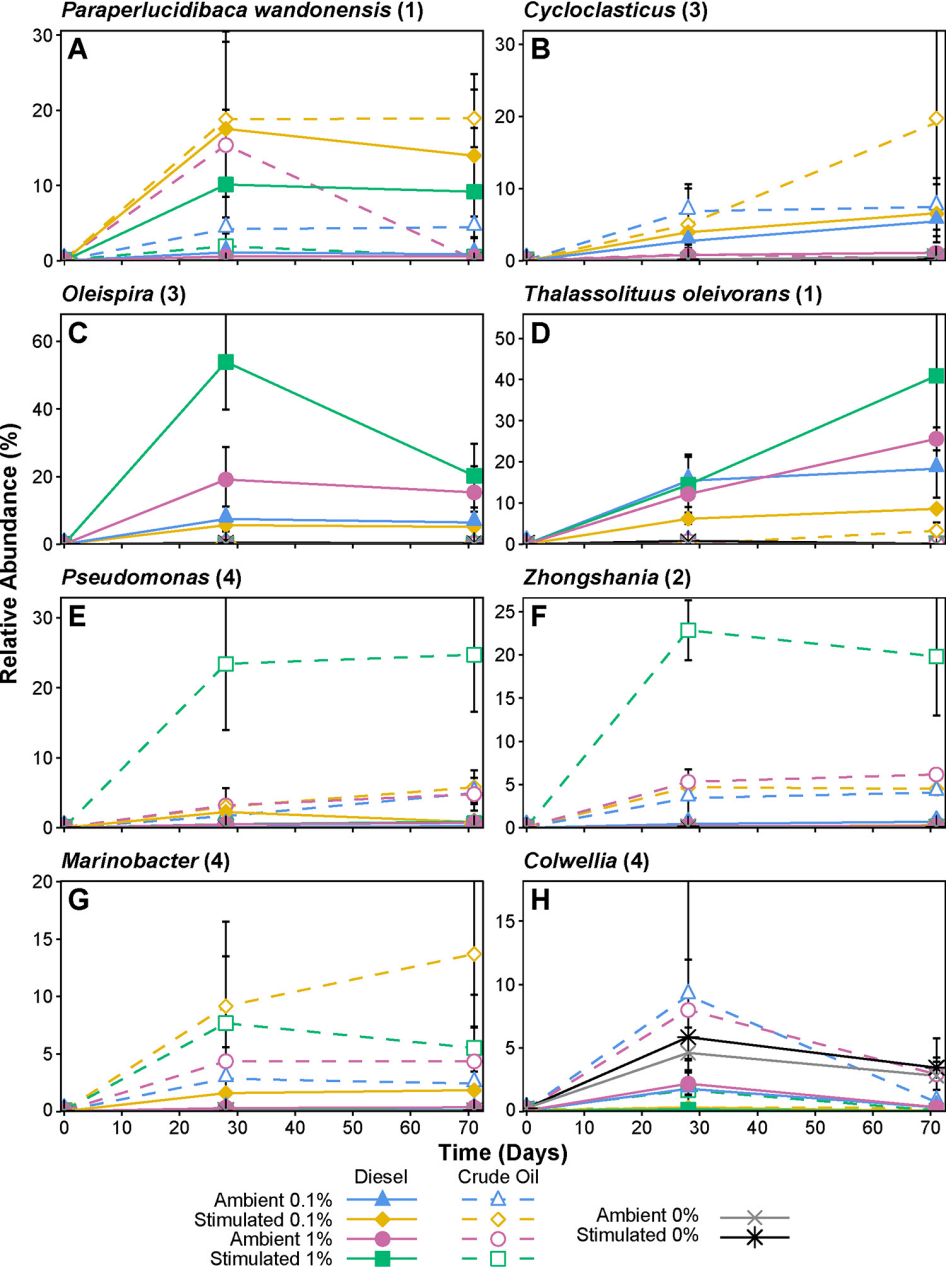
Relative sequence abundance of OTUs belonging to eight different genera, in response to amendment with different diesel and crude oil concentrations (0, 0.1, and 1% [vol/vol]), with (stimulated) or without (ambient) nutrient addition during 71 days of incubation at 4°C. The numbers of OTUs identified in this study belonging to each genus incorporated into the plotted data are shown in parentheses (*Paraperlucidibaca* and *Thalassolituus*, each containing only one OTU, include the closest cultured relatives according to NCBI BLAST searching). Error bars represent standard errors of triplicate microcosms of the same treatment.

Changes in microbial community composition reflect the response to hydrocarbons and biostimulation. Putative HCB, mostly belonging to the *Gammaproteobacteria*, increased in relative abundance within all diesel- and crude oil-amended microcosms (Fig. 6). Across all treatments, *Paraperlucidibaca* increased in relative sequence abundance from <0.1% before incubation to a maximum of 19% by day 28 and maintained a high relative abundance in many diesel- and crude oil-amended treatments at day 71 (Fig. 6A). *Cycloclasticus* increased in abundance from <0.08% to a maximum of 19%, increasing in all 0.1% diesel and 0.1% crude oil amendments until the end of the 71-day incubation (Fig. 6B). Both *Oleispira* (<0.001% up to 54%; Fig. 6C) and *Thalassolituus* (<0.01% up to 41%; Fig. 6D) showed similarly large increases in relative sequence abundance but only in the presence of diesel. Whereas *Oleispira* declined in abundance after 28 days, *Thalassolituus* continued to increase in abundance between 28 and 71 days. Among bacteria responding to crude oil addition, *Zhongshania* (<0.06% up to 23%; Fig. 6F), *Marinobacter* (<0.04% up to 14%; Fig. 6G) and Pseudomonas (<0.04% up to 25%; Fig. 6E) showed the largest increases in relative abundance. None of these genera increased in abundance in unamended controls, whereas within the genus *Colwellia* some OTUs increased in some unamended controls, while others increased in crude oil-amended microcosms (Fig. 6H); Colwellia beringensis OTU_17 was enriched by crude oil amendment, whereas Colwellia psychrerythraea OTU_72 was only enriched among unamended controls (see Fig. S5).

**FIG 7:**
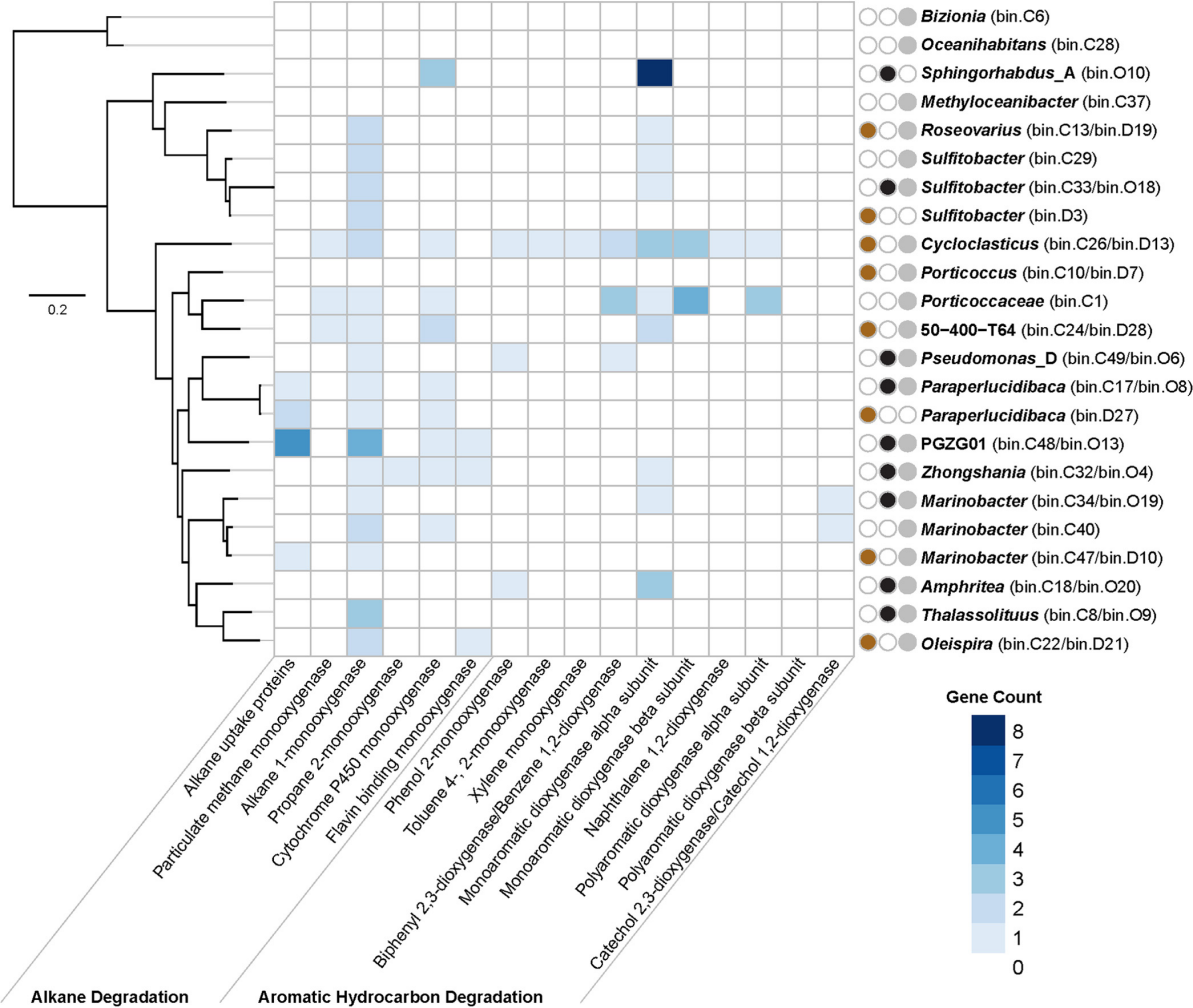
Heatmap representing the gene count of select biodegradation enzymes from MAGs recovered from metagenomic libraries constructed from microcosms amended with diesel (brown) or crude oil (black) and following coassembly of the two (gray). Bin names corresponding to these MAGs are indicated on the right (labeled D, O, and C, respectively) and are arranged according to a maximum-likelihood phylogenomic tree (shown at left) based on 43 concatenated marker genes. The scale bar indicates 0.2 substitutions per nucleotide position. Enzymes containing multiple subunits were counted once if all subunits were present (see Data Set S1). Unpaired mono- or polycyclic aromatic dioxygenase alpha and beta subunits were also counted.

Metagenome-assembled genomes (MAGs) with >70% completeness and <5% contamination were recovered (see Data Set S1) from diesel-amended microcosms (*n* = 8) and crude oil-amended microcosms (*n* = 9). Additional MAGs were also retrieved by coassembly of metagenomes from both conditions (*n* = 20). In total 37 MAGs were retrieved resulting in 23 unique genomes following the removal of duplicates generated by individual and coassemblies (Fig. 7). Enzymes of interest, including monooxygenases, ring-cleaving dioxygenases involved in aerobic activation of alkanes, and mono- or polycyclic aromatic hydrocarbons (40–43), were identified in all 37 MAGs (including duplicates). These metabolic capabilities are intrinsic to aerobic hydrocarbon biodegradation. Complete hydrocarbon degradation pathways among individual genomes do not necessarily confirm the potential ecological effect of a given compound being fully degraded, and full degradation pathways may be shared among complex communities of microbes (44). For this reason, genes associated with initial activation of hydrocarbon degradation were analyzed for this study. Alkane uptake proteins (45) and catechol dioxygenases (46) were also investigated. Genes involved in hydrocarbon degradation were identified in 19 of the 23 unique MAGs (except *Bizionia*, *Oceanihabitans*, *Methyloceanibacter*, and *Porticoccus*; Fig. 7), consistent with hydrocarbon biodegradation potential being a prevalent phenotype among the microbial groups enriched by diesel and crude oil (Fig. 4 and 6). It is interesting that *Porticoccus* MAGs (>98% complete) lacked genes for hydrocarbon degradation, given that members of this genus are believed to degrade hydrocarbons in the phycosphere (47–49).

Alkane 1-monooxygenase genes were widespread among the majority of MAGs, further revealing the potential for alkane degradation in the Labrador Shelf sediment microbiome. This includes *Cycloclasticus*, which is shown here to harbor the potential for degradation of higher alkanes (Fig. 7). MAGs classified as *Paraperlucidibaca*, PGZG01, *Zhongshania*, *Thalassolituus*, and *Oleispira* are also capable of alkane degradation. While aromatic hydrocarbon degradation genes were less prevalent overall, they were abundant in *Cycloclasticus* and *Porticoccaceae* MAGs. Pseudomonas*_*D, Marinobacter psychrophilus, and *Amphritea* MAGs from crude oil amendments also have potential to degrade aromatic hydrocarbons (Fig. 7), consistent with the significantly higher losses of PAHs from these treatments compared to diesel amendments (Fig. 2C and D) and with previous reports of aromatic hydrocarbon biodegradation by *Amphritea* (50–52). MAGs with novel classifications in the Genome Taxonomy Database (GTDB) exhibiting hydrocarbon biodegradation potential included *Sphingorhabdus_*A, 50-400-T64, Pseudomonas*_*D, and PGZG01 (Fig. 7). Most of the MAGs did not include 16S rRNA genes, as has been observed by others (53), but bear taxonomic resemblance to the dominant OTUs that were identified by amplicon sequencing (Fig. 4).

## DISCUSSION

### Diesel and crude oil provoke different members of the Labrador Sea microbiome.

Increases in the relative sequence abundance of key microbial taxa can highlight community succession in response to petroleum contamination. Several taxa showed dramatic increases in relative abundance in response to diesel or crude oil (Fig. 3A, 4, and 6), consistent with Baas Becking’s principle that “everything is everywhere, but, the environment selects” (54). Comparisons revealed 10 OTUs specific to diesel and 18 OTUs specific to crude oil (Fig. 4). Among the six additional indicator OTUs that are shared between the two groups, the most prominent belonged to the *Gammaproteobacteria* and is closely related to Paraperlucidibaca wandonensis (100% sequence identity with OTU_1; Fig. 4A). Other than one study showing that *P. wandonensis* grown at 25°C can degrade Tween (55) (an oleophilic component of dispersants with moieties structurally similar to saturated hydrocarbons), little is known regarding the hydrocarbon degradation capability of *Paraperlucidibaca* spp. In agreement with the prominence of *Paraperlucidibaca* OTU_1 in both diesel- and crude oil-amended microcosms (Fig. 4), *Paraperlucidibaca* MAGs with high completeness (90 to 93%) were recovered from both metagenomic libraries and the coassembly (O8, D27, and C17; Fig. 7). All three MAGs shared >99.7% average nucleotide identity (ANI), indicating the same population was enriched by diesel and crude oil (Fig. 7). The only published *Paraperlucidibaca* genome (*P. baekdonensis*; GCA_003387535.1) shares ∼81% ANI with these MAGs, demonstrating that the Labrador Sea *Paraperlucidibaca* is a separate clade that expands the genomic diversity of this genus. These MAGs contained alkane 1-monooxygenase and cytochrome P450 monooxygenase genes involved in alkane activation, as well as genes encoding uptake proteins that facilitate alkane transfer to the inside of the cell (45), whereas enzymes involved in aromatic hydrocarbon biodegradation were not detected (Fig. 7). This is consistent with *Paraperlucidibaca* being involved in the degradation of alkanes in diesel and crude oil, resulting in its dramatic enrichment, most notably in the presence of nutrients (Fig. 4 and 6). The genus *Paraperlucidibaca* was proposed in 2011 for isolates with lower temperature optima than *Perlucidibaca* spp. (56). Results presented here suggest *Paraperlucidibaca* spp. also include previously unrecognized capabilities for low temperature alkane biodegradation.

Relatives of *Cycloclasticus spirillensus* (95.4% sequence identity with OTU_8; Fig. 4A) were also enriched in both diesel and crude oil amendments (Fig. 6). Unlike *Paraperlucidibaca*, members of the genus *Cycloclasticus* are well-known HCB (19, 57). *Cycloclasticus* MAGs were retrieved from diesel-amended microcosms and exhibited broad potential for hydrocarbon degradation with genes involved in metabolizing aromatic and aliphatic hydrocarbons (Fig. 7). Whereas the former is well known in *Cycloclasticus* (58), members of this genus were only recently shown to be able to degrade alkanes (59, 60). The MAGs obtained here share 76% ANI with *Cycloclasticus* strain TK-8 (confirmed as Cycloclasticus pugetii; https://bacdive.dsmz.de/) isolated from oil slicks of the DWH spill and found capable of degrading aliphatic hydrocarbons (specifically *n*C_14_ and *n*C_15_) in Macondo oil (60). Similarly, these MAGs share 75% ANI with *Cycloclasticus* enriched from a temperate (6 to 8°C) marine channel on dilbit (a mixture of light and heavy petroleum compounds) that contains *alkB* (alkane hydroxylase responsible for aerobic alkane degradation) and *ladA* (a key gene specific to long-chain alkane degradation) (61). Duplicate *Cycloclasticus* MAGs (C26 and D13, 99.9% ANI; Fig. 7) from the Labrador Sea harbor genes for initiating the degradation of short-chain alkanes (e.g., particulate methane monooxygenase genes likely involved in fixing carbon derived from C_2_–C_4_ alkanes [59]; Fig. 7) and long-chain alkanes (e.g., alkane and cytochrome p450 monooxygenase genes; Fig. 7).

Compared against all published *Cycloclasticus* genomes, the highest match for these MAGs shares 89.4% ANI (*Cycloclasticus* sp002292375 and GCA_002292375.1), thus indicating a separate clade and further revealing the prevalence of alkane degradation within the genus. All alkane 1-monooxygenease genes from *Cycloclasticus* MAGs were compared between this study and that of Schreiber et al. (61), revealing a maximum amino acid identity of 77%. Not only is the potential to degrade alkanes broad among different *Cycloclasticus* spp., but these genes are also genetically diverse within the genus. *Cycloclasticus* may play a larger role in the degradation of alkanes in the marine environment than previously thought, an important consideration given that gaseous and other alkanes are released from natural hydrocarbon seeps (62, 63) and from spills like the DWH (64–66) where *Cycloclasticus* was heavily enriched (67). Whether *Cycloclasticus* were responsible for higher alkane degradation in the Labrador Sea microcosms is not clear, given that many other alkane degraders were also present (Fig. 7). On the other hand, of the 23 unique MAGs recovered, *Cycloclasticus*, along with *Porticoccaceae*, encoded the most extensive suite of aromatic hydrocarbon biodegradation genes, with 10 other MAGs exhibiting potential to degrade these compounds to a lesser degree (Fig. 7). The enrichment pattern and genomics of cold-adapted *Cycloclasticus* in the Labrador Sea provide additional evidence that members of this genus are versatile hydrocarbon degraders in low temperature marine environments.

The microbial response specific to diesel amendment (Fig. 4B) featured enrichment of *Oceanospirillales* closely related to Oleispira antarctica (100% sequence identity with OTU_4), Oleispira lenta (99.4% sequence identity with OTU_163) and Thalassolituus oleivorans (100% sequence identity with OTU_3). These genera are known to encompass alkane degraders (68–71) and were especially prevalent in 1% diesel-amended microcosms (Fig. 6C and D), where extensive mineralization (Fig. 2E) was driven primarily by alkane degradation (Fig. 2A and C). *Oleispira* spp. include psychrophiles that respond to oil inputs in cold marine settings (72), possibly explaining *Oleispira* responding rapidly to alkanes in diesel at 4°C, followed by *Thalassolituus* (Fig. 6C and D). These and other *Oceanospirillales* lineages were shown to dominate the microbiome within the crude oil plume during the first month of the Deepwater Horizon oil spill (22, 23, 73) (n.b., many of these groups are reclassified as *Pseudomonadales* in the GTDB [74, 75]). MAGs classified as Oleispira antarctica and Thalassolituus oleivorans both include multiple genes encoding alkane 1-monooxygenase (Fig. 7). *Oleispira* and *Thalassolituus* OTUs were much less prevalent in the presence of crude oil (Fig. 4C and Fig. 6C and D), which experienced less alkane biodegradation than diesel (Fig. 2A to D).

Relatives of Zhongshania borealis (98.3% sequence identity with OTU_5) and Zhongshania antarctica (98.9% sequence identity with OTU_1180) (Fig. 4C) were enriched only in the presence of crude oil, two organisms not previously known to degrade hydrocarbons. *Zhongshania* MAGs recovered from crude oil-amended microcosms harbor a suite of alkane degradation, as well as some monoaromatic dioxygenase genes (Fig. 7). *Zhongshania* spp. more distantly related to OTU_5 and OTU_1180 have been shown to degrade aliphatic hydrocarbons (i.e., *Z. aliphaticivorans*) (76) and *Zhongshania* genomes detected in other cold marine oil bioremediation studies show similar genetic potential for alkane and aromatic hydrocarbon degradation (77, 78) as the MAGs from the Labrador Sea (Fig. 7). This potential and the ubiquity of *Zhongshania* in polar regions (76, 79, 80) suggests this lineage may be relevant as an indicator of hydrocarbon biodegradation during monitored natural attenuation of oil spills in cold marine environments. MAGs classified by GTDB-Tk as PGZG01, within the family *Alcanivoracaceae*, were also retrieved from the 0.1% crude oil amendments. The PGZG01 group may correspond to a novel genus with high potential for low temperature marine alkane degradation, as evidenced by four genes for alkane 1-monooxygenase and five pairs of genes encoding alkane uptake proteins (Fig. 7). An interesting observation in the sediment incubations conducted here is that *Zhongshania* and PGZG01, together with *Paraperlucidibaca*, likely contributed to the biodegradation of alkanes from crude oil by outcompeting well-known *Oleispira* and *Thalassolituus* at 4°C. Understanding subtle differences in microbial responses to diesel and crude oil is a necessary precursor for several bioremediation strategies (81) and will help inform effective responses to future spills.

### Biostimulation promotes biodegradation and microbial community turnover at 4°C.

Naturally low concentrations of nitrogen and phosphorus in the marine environment combined with low temperatures in regions like the Labrador Sea are constraints governing the biodegradation of spilled hydrocarbons. This study showed how diesel and crude oil spills in concentrations of 0.1 and 1% (vol/vol) rapidly deplete the ambient nutrient supply (see Fig. S2), thereby limiting the extent of biodegradation that is possible. As a marine oil spill response measure, biostimulation is considered worthwhile if the biodegradation rate can be doubled (82). Nutrient application resulted in respiration rates that more than doubled during the first few weeks of incubation (Fig. 2G and H), coinciding with accelerated removal of alkanes and PAHs (Fig. 2C and D). Respiration rates coupled to diesel and crude oil degradation declined after the first 28 days of 4°C incubation (Fig. 2G and H). It has been suggested that nutrient biostimulation may be most effective early on, as observed here, and that following an initial pulse of activity in response to nutrient addition, natural attenuation without additional biostimulation may be a preferable strategy (83, 84).

Different genera were enriched in response to different diesel and/or crude oil treatments, with or without nutrient stimulation (Fig. 4 and 6). Shifts in microbial community composition and α-diversity (Fig. 3) are consistent with other studies showing that oil contamination facilitates growth and competition leading to less diverse communities (21, 77, 78, 85). Sun et al. (86) reported that microbial communities stimulated with 2% (wt/vol) crude oil, much higher nutrient concentrations, and incubated at 30°C were not significantly different from those treated with oil alone; nutrients influenced the succession of bacterial communities, while actual differences were more closely related to the addition of crude oil. In this more realistic marine oil spill scenario using Labrador Sea sediments incubated at cold *in situ* temperature, comparing ambient and biostimulated nutrient treatments showed that nutrient addition similarly influences community succession and timing whereas responding populations were influenced more by the petroleum product. Major community shifts in response to biostimulation occurred within the first 28 days when rates of CO_2_ generation were greatest (Fig. 2E and F). Nutrient stimulated treatments exhibited the most significant change in Bray-Curtis dissimilarity during this period (Fig. 3A; see also Data Set S3) and potentially allowed HCB to establish earlier as evidenced by the shorter apparent lag in respiration among diesel amendments (Fig. 2E).

Biostimulation enhanced the loss of hydrocarbons in 0.1% diesel and crude oil amendments (Fig. 2A to D) but had almost no effect in the presence of 1% diesel or crude oil, with the exception of alkanes in diesel being consumed (Fig. 2A). Greater alkane losses and CO_2_ production (Fig. 2E and F) coupled with low PAH losses among nutrient-stimulated 1% diesel treatments were likely due to a large bloom in biomass (as evidenced by DNA yields; see Fig. S4) consisting of alkane-degrading bacteria (i.e., *Thalassolituus*, *Oleispira*, and *Paraperlucidibaca*; Fig. 4) and a lack of succession to aromatic hydrocarbon degraders (21). *Cycloclasticus* (OTU_8) was one of the only OTUs with corresponding potential for aromatic hydrocarbon biodegradation (Fig. 7) to be enriched in the presence of diesel, but its low abundance in 1% diesel amendments by day 71 suggests community succession did not occur (Fig. 4A). This points to the concentration of spilled petroleum playing a role in the timing of community succession even when nutrients are not limiting. Among 1% crude oil amendments there was no difference in alkane or PAH losses between ambient nutrient and stimulated treatments, despite large increases in CO_2_ in the latter (Fig. 2E and F). Compounds other than the measured alkanes and PAHs may account for this activity, and it cannot be ruled out that increases in CO_2_ in these nutrient-stimulated crude oil microcosms (Fig. 2F) are due to degradation of necromass owing to toxicity that 1% crude oil exerts on the community as a whole. Toxic components of crude oil that are not present in diesel may include certain monoaromatics, *n*-alkanols, and terpenoids (87), potentially explaining these results. High levels of certain compounds may strongly inhibit bacterial growth, resulting in poor biodegradation efficiency (88, 89), potentially rendering nutrient addition ineffective among 1% crude oil treatments.

Whether biostimulation should be used in response to spilled hydrocarbons will be situationally dependent, determined by the environment, the type of petroleum product released, and its volume and concentration. Nutrient application along shorelines after the Exxon Valdez oil spill proved successful at enhancing biodegradation (9, 84, 90, 91). Areas of upwelling or terrestrial runoff where nutrient concentrations may be naturally elevated could further mitigate hydrocarbon pollutants in the event of a spill, and such areas should be identified when planning shipping routes through remote environments. Whereas chemical dispersants are used in response to oil spills in open water (92) where biostimulation would result in rapid dilution of applied nutrients, dispersants will have limited effectiveness in instances where physical factors result in pooling of the petroleum contamination. The concentrations of diesel and crude oil employed in the low-temperature Labrador Sea sediment experiments presented here are higher than what would be encountered following dispersant application in open water (28) and are more realistic in the context of pooling associated with shorelines (9, 84), sea ice floes (93), or upon export to the seabed via sedimentation and accumulation (94–96). Residual hydrocarbons in Gulf of Mexico sediments in the aftermath of the DWH spill were greater than 100 ppm and represent a continuing issue due to long-term persistence and potential redistribution (97). Biostimulation technologies that target hydrocarbons in sea-floor sediments may prevent prolonged exposure of benthic organisms such as economically important fish in the Gulf of Mexico or those essential to the traditional livelihoods of communities along the coast of the Labrador Sea. This study demonstrates the potential for low-temperature biodegradation by Labrador Sea microbial communities and the likelihood for nutrient biostimulation to enhance this response.

## MATERIALS AND METHODS

### Labrador Shelf sediment collection.

Sediment samples were collected in 2015 aboard the Canadian Coast Guard Icebreaker *Amundsen* at 58°55.609N, 62°09.326W in the Labrador Sea (Fig. 1) from water depths ranging from 141 to 145 m (see Table S1). Surface sediment was collected from within the top 0 to 5 cm of three separate box core casts (see Table S1) and immediately stored in sterile plastic bags at 4°C on board the ship. Upon return to the University of Calgary, bulk sediments remained stored at 4°C for several months until microcosms were established.

### Hydrocarbon- and nutrient-amended microcosm incubations at 4°C.

Triplicate microcosms were established from separate sediment samples from the triplicate box core casts. Diesel used in this experiment was obtained from a local gas station where common diesel is comprised of 75% saturated hydrocarbons and 25% aromatic hydrocarbons with an average formula of C_12_H_23_. Crude oil used in this experiment came from the Macondo oil reservoir in the Gulf of Mexico, and similar to crude oils produced offshore Newfoundland and Labrador, is a light, nonviscous oil with initial API gravity of 35° (98). Microcosms were established in 160-ml glass serum bottles with a 1:4 ratio of sediment to medium and incubated at 4°C to simulate ambient benthic temperatures. Artificial seawater (ASW) medium (99) was added as 40 ml except for the sediment-free control bottles which had the sediment volume replaced by using 50 ml of medium. Sodium sulfide was omitted from the ASW to promote oxic conditions in the microcosms. All bottles were hermetically sealed and a slight overpressure was established using filter-sterilized (0.2 μm) air. For diesel amendments, despite adding 20-ml sterilized air to provide ample oxygen for the duration of the experiment, one treatment group (nutrient stimulated 1% [vol/vol] diesel, all three replicates) became anoxic (see Fig. S1E). The initial volume of injected air for subsequent crude oil amendments was therefore increased to 60 ml to prevent the establishment of anoxic conditions (see Fig. S1F).

Four treatment groups were established for both diesel and crude oil amendments using two batches of ASW, one with a low level of N and P (15 μM NH_4_Cl and 2 μM KH_2_PO_4_) to mimic regional bottom water concentrations (100), and one with higher levels (4.67 mM NH_4_Cl and 1.47 mM KH_2_PO_4_), to simulate nutrient biostimulation. The lower concentrations were selected to observe how rapidly ambient nutrient levels deplete after the introduction of hydrocarbons, thus impacting rates of hydrocarbon biodegradation. The higher nutrient concentrations satisfy the Redfield ratio by providing much more N and P than would be required to degrade the volumes of diesel and crude oil added in order to demonstrate how nutrient addition can further stimulate hydrocarbon degradation. Either diesel or crude oil was added at concentrations of 0.1 or 1.0% (vol/vol) of the combined sediment and medium (i.e., 50 or 500 μl, respectively). These levels were selected to determine whether concentration influenced microbial community composition and response, with 1% representing a pooled oil scenario and 0.1% representing a more dispersed scenario. Densities of Macondo crude oil and diesel are 0.84 and 0.85 g/cm^3^, respectively; thus, their concentration per gram of wet sediment in incubations was estimated at 840 to 860 μg g^−1^ in 0.1% (vol/vol) incubations, and 8,400 to 8,600 μg g^−1^ in 1% (vol/vol) incubations. For each diesel or crude oil concentration, a parallel set of microcosms was prepared and immediately frozen at −20°C for chemical analyses of hydrocarbons to define preincubation conditions. The experimental setup included sediment-free controls, killed (autoclaved) controls, and unamended (i.e., no diesel or crude oil) controls. Killed controls were autoclaved once at the onset of the diesel experiment, and three times at the onset of the crude oil experiment. All microcosms were incubated at 4°C and monitored for 71 days.

### Gas chromatography measurements.

Concentrations of CO_2_ and O_2_ in all microcosms were monitored at 0, 11, 28, and 71 days by injecting 1 ml of headspace gas into an Agilent 7890B gas chromatograph (GC). Separation of CO_2_ was performed on a GC packed Hayesep N packing column (stainless steel tubing, 0.5-m length × 1/8″ OD, 2-mm internal diameter [ID]; mesh size, 80/100), followed by a similar column at 1.83-m length. Separation of O_2_ was performed on a GC packed MolSieve 5A packing column (UltiMetal tubing, 2.44 m, 1/8″ OD, 2-mm ID; mesh size, 60/80). Helium was applied as the carrier gas at a flow rate of 21 ml/min. Gases were detected by a thermal conductivity detector (TCD-3) set to 200°C. The oven temperature program was set to 105°C for 5 min.

### Hydrocarbon analysis by gas chromatography-mass spectrometry.

Hydrocarbons were extracted from diesel- and crude oil-amended microcosms that had been frozen at −20°C after the 71-day incubation period, as well as from duplicate treatment microcosms immediately frozen at day 0. After thawing, hydrocarbons were extracted via dissolution in dichloromethane (DCM) with the use of sonication. Extracts were run through a column of sodium sulfate to remove any residual water, and DCM was evaporated to a final concentrated volume of 1 ml. Extracts were injected for GC-MS using an Agilent 7890B GC and 5977A quadrupole mass-selective detector. Samples were injected as a 1 μl aliquot and separation was performed on a HP-5 MS capillary column (Agilent, 30 m × 0.25-mm ID × 0.25-μm film thickness) with a splitless injector (pulsed mode) at 250°C. High-purity helium (99.999%) was applied as the carrier gas at a flow rate of 1 ml/min. The temperature program was as follows: 40°C held for 5 min, followed by heating to 325°C at 4°C/min and finished with an isothermal hold for 15 min at 325°C. The mass spectrometer acquired data in a combined full scan/selected ion monitoring (SIM-SCAN) mode. An ion abundance of 500,000 to 1,000,000 was required; therefore, certain samples were concentrated or diluted with DCM to fit that range and rerun. Using Agilent MSD ChemStation E.02.02.1431, alkane (*n*-alkanes C_12_-C_25_ and pristane), and PAH (phenanthrene, biphenyl, naphthalene, methylnaphthalene (MN; 1-MN and 2-MN), dimethylnaphthalene (DMN; 2,6+2,7-, 1,3+1,7-; 1,6-; 1,4+2,3-; and 1,5-DMN), and trimethylnaphthalene (TMN; 1,3,7-, 1,3,6-, 1,2,4-, 1,2,5-, and 1,2,3-TMN) fragments were identified and ion peaks integrated. GC-MS measurements were used as a metric to assess degradation between the different treatment conditions, with the same compounds compared following amendment with diesel or crude oil.

### Identification of conserved markers for determining biodegradation.

Common diagnostic isomer ratios (see Table S2) were used for confirmation of both alkane (*n*-C_17_/pristane) (101), and PAH (2-methylphenanthrene/1-methylphenanthrene) (102) degradation. Biodegradation was further determined as the preferential loss of select compounds compared against chemical constituents understood to be recalcitrant to degradation, described below. Depletion of target compounds in diesel or crude oil was calculated using the following equation (103), where *A_s_* and *C_s_* are the concentrations of the target compound and conserved compound in the 71-day incubation, respectively, and *A_0_* and *C_0_* are the concentrations at day 0:

$$\%\text{ loss }=\;\left[\left(\left(A_{0}/C_{0}\right)\;–\;\left(A_{s}/C_{s}\right)\right)/\left(A_{0}/C_{0}\right)\right]\;\times \text{ 1}00$$

In crude oil-amended samples, integrated areas were normalized against the area of the conserved compound C_30_-hopane [17α(H),21β(H)-hopane]. Hopanes are common among crude oils and very resistant to biodegradation making them an ideal internal standard for assessing the biodegradation of other oil compounds (104). Since hopanes are not present in diesel, 1,3+3,9+2,10+3,10-dimethylphenanthrene was used as the conserved compound for normalization to assess biodegradation in diesel-amended microcosms. Methylated phenanthrenes have previously been used as conserved markers in the biodegradation of diesel (105).

### 16S rRNA gene amplicon sequencing and analysis.

DNA was extracted from frozen microcosm sediment slurry samples taken at days 0, 28, and 71 using the Qiagen DNeasy PowerLyzer PowerSoil kit and manufacturer instructions. Immediately prior to bead beating, 300-μl samples were heated to 70°C for 10 min to assist with cell lysis. The PowerLyzer 24 bead beater was operated at 6,000 rpm for 40 s. At the final extraction step, 50 μl of elution buffer heated to 50°C was used. Extracted DNA was quantified using a Qubit fluorometer and stored at −20°C. For each round of DNA extraction, one procedural blank consisting of 300 μl extraction buffer was included. PCR of 16S rRNA genes used Illumina modified primers 341F (S-*-Univ-0341-b-S-17) and 806R (S-d-Bact-0787-b-A-20), as described elsewhere (106, 107), in 25-μl reactions (contents: 12.5 μl of 2× KAPA HiFi Hot Start Ready Mix, 2.5 μl of each primer [1 mM], 1 to 10 μl of template DNA, and sterile water). Using an Eppendorf thermal cycler the double-stranded DNA was denatured at 95°C for 3 min, followed by 25 cycles of denaturation at 95°C for 30 s, annealing at 56°C for 45 s, extension at 72°C for 1 min, and then a final extension at 72°C for 5 min. PCR products were run on a 1% agarose gel stained with SYBR green (Thermo Fisher Scientific) to confirm amplicons of the expected length and no amplicons in negative controls. For each sample, three separately amplified PCR products were pooled to reduce bias associated with early cycle PCR-induced errors and to minimize PCR drift (108). PCR products were purified using AMPure XP magnetic beads, barcoded with Illumina index primers as described previously (109), quantified using the Qubit fluorometer, pooled by normalizing for the concentration of DNA among samples, then sequenced using an in-house Illumina MiSeq (Energy Bioengineering and Geomicrobiology group, University of Calgary). Pooled libraries were sequenced using a 600 cycle MiSeq v3 reagent kit, producing 2 × 300-bp paired-end reads.

Sequence files (FASTA) were uploaded and analyzed using MetaAmp version 2.0 (110), a pipeline for processing of SSU rRNA genes using UPARSE, Mothur, and the SILVA database. Parameters were set to assemble pair-end reads and cluster them into OTUs according to a 97% sequence identity cutoff. The minimum length overlap of pair end reads was selected as 80 bp, with a maximum of 8 bp (10%) mismatches allowed in the overlap region. Default quality filtering options were used (maximum number of differences to the primer sequence, 0; maximum number of expected errors, 1; trimmed amplicon length, 350). The generated OTUs were given taxonomic assignments by MetaAmp via sequence comparisons with the SILVA database. Prokaryotic 16S rRNA genes and that of chloroplasts are homologous; therefore, amplified chloroplast 16S rRNA genes were removed from these files to allow for microbial analysis exclusively.

### Metagenomic sequencing and analysis.

Triplicate DNA extractions were pooled from nutrient stimulated microcosms incubated for 71 days with 0.1% (vol/vol) diesel or crude oil. DNA was mechanically sheared using a Covaris S220 Focused-ultrasonicator (Covaris, Inc., Woburn, MA) to generate ∼450-bp fragments. Libraries were constructed using the NEBNext Ultra II Library Prep kit (New England Biolabs, Inc., Ipswich, MA) and sequenced on an Illumina NovaSeq S4 (PE 150 × 150; Illumina, Inc., San Diego, CA) at the Center for Health Genomics and Informatics, University of Calgary. Quality filtering of raw paired-end reads was performed using BBDuk with the parameters *qtrim = rl trimq = 20 minlength = 30 entropy = 0.5* (http://jgi.doe.gov/data-and-tools/bb-tools/) to remove Illumina adapters, phiX sequences, and reads shorter than 30 bp. Post-QC read quality was evaluated using FastQC (http://www.bioinformatics.babraham.ac.uk/projects/fastqc/). Resulting metagenome libraries for diesel- and crude oil-amended incubations were 30.1 and 31.8 million paired-end reads, respectively.

Libraries were assembled individually and coassembled using MEGAHIT v1.2.2 (111) with the parameters *-min-contig-len 500* to ensure contigs longer than 500 bp. Quality filtered reads were mapped to both assemblies and the coassembly using BBMap with default parameters. This was followed by generation of sequencing depth profiles using the *jgi_summarize_bam_contig_depths* tool provided with MetaBAT2 (112), which provided differential coverage values to improve binning. Metagenome-assembled genomes (MAGs) were binned using MetaBAT2 with default parameters and then evaluated for completeness and contamination using the *lineage_wf* workflow of CheckM v1.0.11 (113). MAGs were assigned taxonomy using the *classify_wf* workflow of GTDB-Tk v1.3.3 (114). The marker gene concatenated alignment of the selected MAGs generated by CheckM was used to construct a phylogenomic tree using FastTree v2.1.5 (115). To assess the presence of hydrocarbon degradation genes and pathways, a comprehensive annotation profile for each assembly was generated using MetaErg (116) with parameters *–mincontiglen 500 –minorflen 180 –gtype meta –gcode 11*. The annotation profile contains the prediction and abundances of protein-coding genes. Proteins of interest identified from MetaErg outputs were verified using NCBI Protein BLAST (117) and presented in Data Set S1. Duplicate MAGs were obtained by running individual assembly and coassemblies. Instead of eliminating duplicates with dereplication, both were investigated for protein-coding genes owing to some duplicates having different levels of completeness. FastANI (118), with minimum fragment length set to 1,000 bp and other parameters set to default, was used to compare the percent average nucleotide identity (ANI) of select MAGs and published genomes. To identify potential contamination in contigs from select MAGs (*Cycloclasticus*, *Paraperlucidibaca*, and *Zhongshania*), MAGPurify (119) modules “gc-content” and “tetra-freq” were run to identify any contigs with outlier nucleotide composition based on GC content and tetranucleotide frequencies, respectively. No contamination was found among the investigated genomes.

### Data analysis.

Data for gas values, hydrocarbon losses, and 16S rRNA gene comparisons were tested for significance using analysis of variance (ANOVA) and tested for normality using ANOVA residuals with the Shapiro-Wilk test (see Data Set S2). Significant ANOVAs were further analyzed with *post hoc* multiple pairwise comparisons using the Tukey HSD test. Non-normally distributed data (*P* value of Shapiro-Wilk normality test, <0.05) were analyzed using the Kruskal-Wallis rank sum test, followed by the pairwise Wilcox test. A *P* value of 0.05 was used as the cutoff for statistical significance. All statistical analyses were performed using R (120) and test results are presented in Data Set S2.

Microbial community analysis was performed in R using output files generated by MetaAmp 2.0 and R scripts available online (https://jkzorz.github.io/blog/). Community analyses comparing OTU or genus abundance between treatments were generated using full library sequence reads, ranging from 3,793 to 136,349 reads after chloroplast removal (see Table S3). Community analyses involving α- and β-diversity comparisons between treatments (NMDS, Shannon Index, and observed OTUs) used MetaAmp sequence data rarefied to the lowest library sequence value (i.e., 4,677). OTU correlations were performed using the Spearman’s rank correlation coefficient. Bray-Curtis dissimilarity was used to quantify the differences between microbial communities of treatment and control groups based on 16S rRNA gene libraries and visualized using NMDS implemented by the R package “vegan” (121) to assess changes in community composition in response to hydrocarbon- and nutrient-amendment over time. The ANOSIM function in vegan was used to test for statistically significant differences between designated groups (see Data Set S3). The function “envfit” was used to plot experimental vectors and factors (nutrient concentration, petroleum product, and hydrocarbon concentration), their correlation with the ordination, and *r*^2^ and significance values. The similarity percentage function “simper” (122) was used to identify the OTUs that contributed most to community dissimilarities identified through ANOSIM by performing pairwise comparisons of groups and finding the average contributions of each OTU to the average overall Bray-Curtis dissimilarity (see Data Set S3). OTUs that contributed to at least 50% of the dissimilarity were examined using the R package “indicspecies” (123) to determine those significantly associated with a given treatment (see Data Set S4). The taxonomy of key OTUs was determined by comparing the representative sequence with closest cultured relatives in the GenBank database following NCBI BLAST searching of the RNA reference database (117).

### Data availability.

All raw 16S rRNA gene sequence reads generated for this study have been submitted to NCBI’s Sequence Read Archive (SRA) and are available within the BioProject PRJNA686385 (accession numbers SRX9713174 to SRX9713253). Raw metagenome sequence reads have been submitted to SRA (accession numbers SRX10509741 to SRX10509742), and MAGs have been submitted as a Whole Genome Shotgun (WGS) project (accession numbers JAGPUQ000000000 to JAGPWA000000000), all under the same BioProject.
