# Supplementary material for: Diesel and Crude Oil Biodegradation by Cold-Adapted Microbial Communities in the Labrador Sea
Source: Appl Environ Microbiol. 2021 Sep 28;87(20):e00800-21. doi: 10.1128/AEM.00800-21 (PMC8478444; doi:10.1128/AEM.00800-21)
Supplement: Supplemental file 1 — Nutrient analysis method, Tables S1 to S3, Fig. S1 to S5. Download AEM.00800-21-s0001.pdf, PDF file, 1.0 MB [file aem.00800-21-s0001.pdf]

Supplementary Material

Nutrient analysis method

Ammonium depletion was monitored using the O-phthalaldehyde (OPA) colorimetric assay (1-2) after 0, 28, and 71 days incubation. 2 mL of slurry was removed from microcosms after vigorous shaking, centrifuged, and the supernatant frozen at -20°C until measured. OPA was used in combination with sodium sulfite and sodium tetraborate to bind with NH_4^+ using methods allowing for detection of small concentrations. Thawed supernatant (0.5 mL) was mixed with OPA working reagent at a 1:4 ratio and incubated for 3 hours. Spectrophotometry using a Synergy™ HTX Multi-Mode Microplate Reader set to 410 nm was used for NH_4^+ concentrations > 2 µM. Fluorometry using an Agilent Cary Eclipse Fluorescence Spectrophotometer was required for NH_4^+ concentrations < 2 µM. Standard curves of absorbance and concentration were created to calculate the concentration in measured samples.

References

1. Goyal SS, Rains DW, Huffaker RC. 1988. Determination of ammonium ion by fluorometry or spectrophotometry after on-line derivatization with o-phthalaldehyde. Anal Chem, 60:175-179.
2. Holmes RM, Aminot A, Kérouel R, Hooker BA, Peterson BJ. 1999. A simple and precise method for measuring ammonium in marine and freshwater ecosystems. Can J Fish Aquat, 56:1801-1808.

Supplementary Tables

TABLE S1: Sediment sample metadata.

Core	Collection Date (YYYY-MM-DD)	Geographic Location	Latitude	Longitude	Sample Material	Depth (mbss[1])	Sediment Depth (cmbsf[2])	Collection Method
**1**	2015-10-27	Labrador Sea	58°55.609 N	62°09.326 W	Sediment	145	0-5	Box Corer
**2**	2015-10-27	Labrador Sea	58°55.694 N	62°09.412 W	Sediment	141	0-5	Box Corer
**3**	2015-10-27	Labrador Sea	58°55.905 N	62°09.458 W	Sediment	141	0-5	Box Corer

[1] meters below sea surface

[2] centimetres below sea floor

TABLE S2: Common diagnostic isomer ratios used as indicators of biodegradation.

Ratio	Treatment	Diesel	Crude Oil
**C_{17}/Pr[1]**	Day-0 Pre-treatment	2.37 ± 0.03	2.17 ± 0.01
	Day-71 Ambient / 0.1%	1.54 ± 0.14	2.10 ± 0.03
	Day-71 Stimulated / 0.1%	0.95 ± 0.27	1.93 ± 0.05
	Day-71 Ambient / 1%	2.24 ± 0.04	2.21 ± 0.02
	Day-71 Stimulated / 1%	1.34 ± 0.03	2.22 ± 0.03
**2MN/1MN[2]**	Day-0 Pre-treatment	1.80 ± 0.003	1.56 ± 0.01
	Day-71 Ambient / 0.1%	1.30 ± 0.15	1.11 ± 0.06
	Day-71 Stimulated / 0.1%	1.12 ± 0.16	1.01 ± 0.12
	Day-71 Ambient / 1%	1.69 ± 0.12	1.54 ± 0.02
	Day-71 Stimulated / 1%	1.79 ± 0.01	1.45 ± 0.04

Mean ratios of [1]*n*-C_{17}/pristane (C_{17}/Pr) and [2]2-methylnaphthalene/1-methylnaphthalene (2MN/1MN) with standard error, from diesel and crude oil at 0 and 71 days of incubation at 4°C.

TABLE S3: 16S rRNA sequence read number by sample.

Sample ID[1]	Original Sequence Reads	Sequence Reads (Chloroplasts Removed)	Rarefied Sequence Reads	Rarefied Sequence Reads (Chloroplasts Removed)
Range	(4677 - 137595)	(3793 - 136349)	4677	(3501 - 4657)
D_T0_1_0_H	6364	5155	4677	3768
D_T0_2_0_H	13348	11440	4677	4018
D_T0_3_0_H	21472	19020	4677	4123
D_T0_1_1_H	29345	25033	4677	3989
D_T0_2_1_H	44843	40497	4677	4218
D_T0_3_1_H	8944	8314	4677	4324
D_T28_1_0_L	10531	8630	4677	3841
D_T28_2_0_L	8776	7708	4677	4091
D_T28_3_0_L	12245	10639	4677	4055
D_T71_1_0_L	7520	6203	4677	3838
D_T71_2_0_L	13278	11745	4677	4139
D_T71_3_0_L	17251	15105	4677	4073
D_T28_1_0_H	15564	13558	4677	4085
D_T28_2_0_H	15541	14191	4677	4257
D_T28_3_0_H	10798	9528	4677	4138
D_T71_1_0_H	26216	23642	4677	4215

D_T71_2_0_H	71671	66965	4677	4377
D_T71_3_0_H	20761	19042	4677	4297
D_T28_1_01_L	39550	39340	4677	3501
D_T28_2_01_L	22505	20813	4677	4337
D_T28_3_01_L	13630	12037	4677	4134
D_T71_1_01_L	10089	9558	4677	4144
D_T71_2_01_L	6102	5786	4677	4422
D_T71_3_01_L	27040	25358	4677	4404
D_T28_1_01_H	30390	29556	4677	4537
D_T28_2_01_H	59855	58462	4677	4567
D_T28_3_01_H	35321	26835	4677	4517
D_T71_1_01_H	10490	9833	4677	4385
D_T71_2_01_H	10744	10613	4677	4621
D_T71_3_01_H	8416	7468	4677	4510
D_T28_1_1_L	4677	3793	4677	3793
D_T28_2_1_L	6511	5739	4677	4096
D_T28_3_1_L	65628	63718	4677	4334
D_T71_1_1_L	14381	13320	4677	4341
D_T71_2_1_L	12667	12293	4677	4547
D_T71_3_1_L	6653	6419	4677	4485
D_T28_1_1_H	28800	28658	4677	4655
D_T28_2_1_H	52680	51957	4677	4619
D_T28_3_1_H	22232	21305	4677	4655

D_T71_1_1_H	11586	11440	4677	4619
D_T71_2_1_H	6691	6586	4677	4618
D_T71_3_1_H	28790	26356	4677	4434
C_T0_1_0_H	20813	18655	4677	4210
C_T0_2_0_H	10337	9747	4677	4415
C_T0_3_0_H	25240	23701	4677	4377
C_T0_1_1_H	42408	36541	4677	4018
C_T0_2_1_H	29548	28007	4677	4428
C_T0_3_1_H	28424	26919	4677	4424
C_T28_1_0_L	23827	22102	4677	4369
C_T28_2_0_L	26189	25227	4677	4527
C_T28_3_0_L	83895	79925	4677	4460
C_T71_1_0_L	11902	11068	4677	4367
C_T71_2_0_L	12896	12440	4677	4511
C_T71_3_0_L	27776	26729	4677	4493
C_T28_1_0_H	35228	33030	4677	4404
C_T28_2_0_H	24451	23579	4677	4524
C_T71_1_0_H	26451	25369	4677	4465
C_T71_2_0_H	30988	30166	4677	4555
C_T28_1_01_L	25990	24011	4677	4315
C_T28_2_01_L	33887	33092	4677	4576
C_T71_1_01_L	35454	33698	4677	4445
C_T71_2_01_L	31625	30999	4677	4585

C_T28_1_01_H	23202	22641	4677	4564
C_T28_2_01_H	23769	21732	4677	4301
C_T28_3_01_H	28960	28627	4677	4630
C_T71_1_01_H	24957	24738	4677	4637
C_T71_2_01_H	26343	26208	4677	4657
C_T71_3_01_H	18314	18224	4677	4650
C_T28_1_1_L	42162	41859	4677	4649
C_T28_2_1_L	44713	43745	4677	4563
C_T28_3_1_L	25836	25182	4677	4560
C_T71_1_1_L	29582	27810	4677	4391
C_T71_2_1_L	21644	21292	4677	4604
C_T71_3_1_L	118420	114834	4677	4557
C_T28_1_1_H	30586	29720	4677	4550
C_T28_2_1_H	29181	28986	4677	4649
C_T28_3_1_H	23981	23813	4677	4653
C_T71_1_1_H	28849	28278	4677	4590
C_T71_2_1_H	137595	136349	4677	4640
C_T71_3_1_H	27068	26909	4677	4652

[1]Sample IDs indicate the petroleum product (D = diesel, C = crude oil), time point (T0, T28, or T71), sediment core (1, 2, 3), hydrocarbon concentration (0, 0.1, or 1% v/v), and nutrient availability (L = low/ambient, H = high/stimulated).

34 **Supplementary Figures**

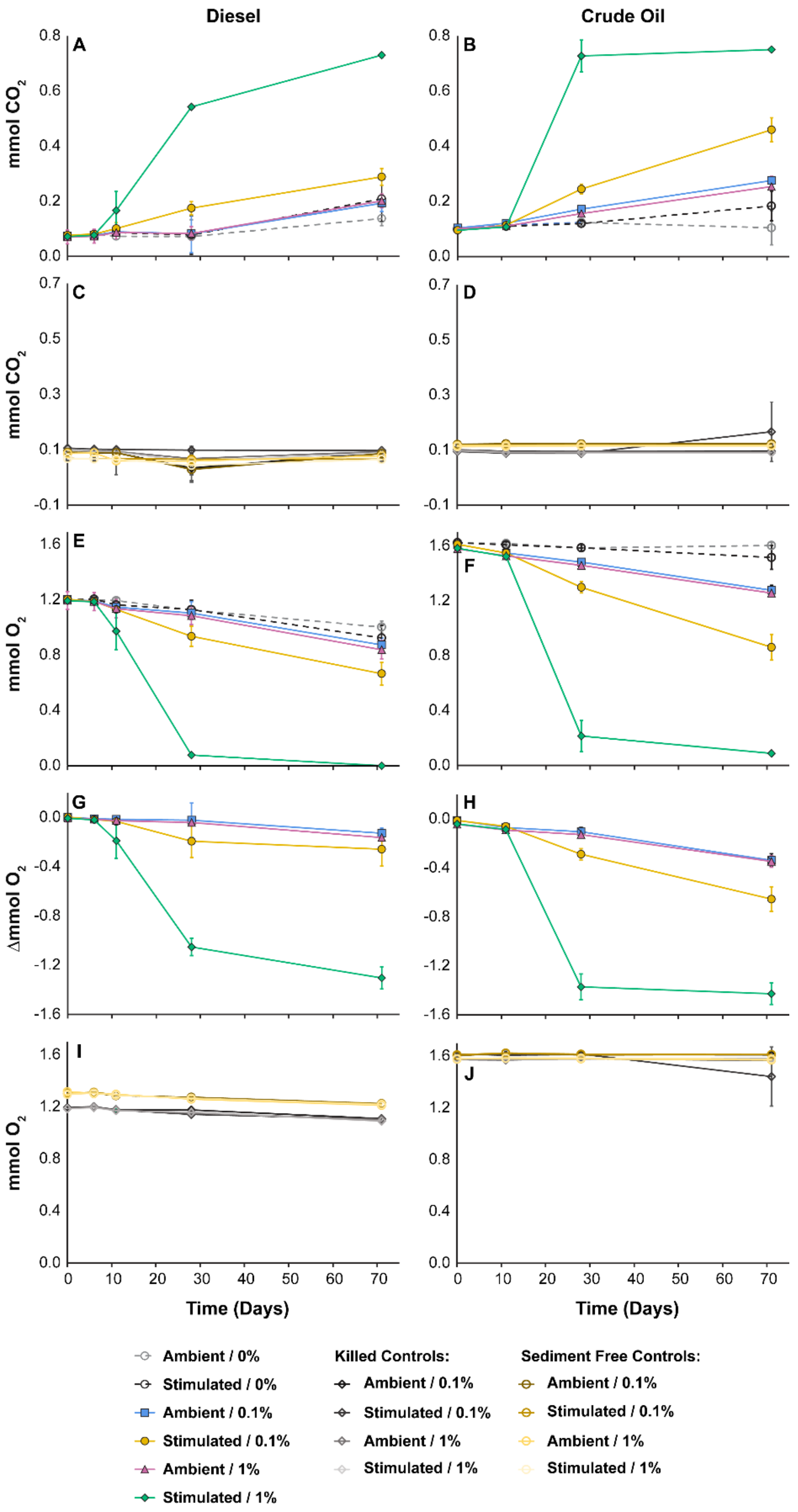

35

FIG S1: Values of CO_2 and O_2 (mmol) within diesel- (left) and crude oil-amended (right) sediment microcosms obtained using GC over 71 days of incubation at 4°C. Data points represent the mean of three replicate microcosms established with three separate sediment core casts with error bars as standard deviation. Panels A-B (CO_2) and E-F (O_2) show absolute values of gases within the headspace of the microcosms, with hydrocarbon amended microcosms (solid lines) and unamended control microcosms (dashed lines). In panels G-H the Δ represents the difference between unamended and amended microcosms, the equivalent for CO_2 is reported in Fig. 2E-F. Panels C-D and I-J present gas data from the sediment free and killed control microcosms, whereby no change was observed over the duration of the incubations. Only one crude oil killed replicate (Stimulated / 0.1%) was found to be contaminated, showing elevated CO_2 and reduced O_2 after 28 days of incubation.

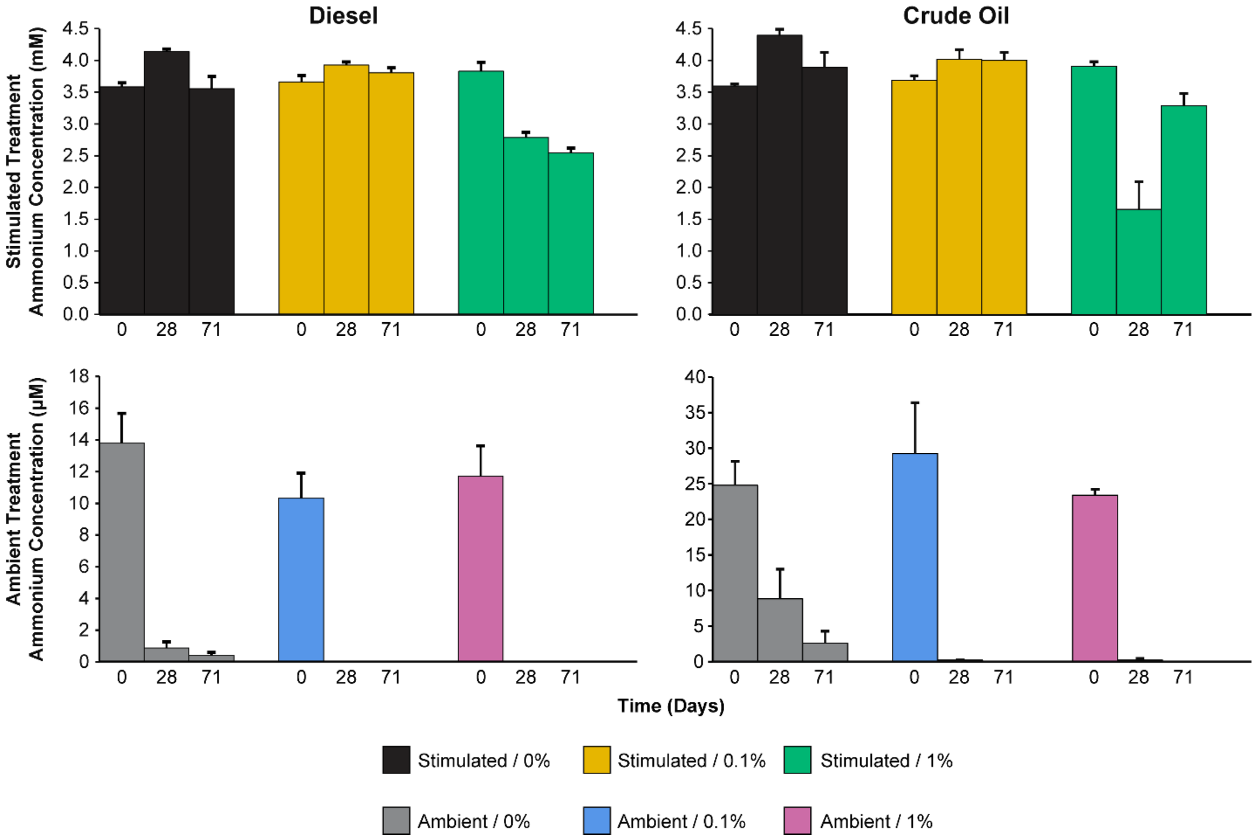

FIG S2: Ammonium concentrations within treatments and controls at 0, 28, and 71 days of incubation within diesel (left) and crude oil (right) amended microcosms. Triplicate microcosms are averaged with error bars as standard error. In both diesel and crude oil microcosms, ambient nutrient treatments were rapidly depleted of ammonium. Note that the concentrations are reported differently for ambient (µM) and nutrient stimulated (mM) treatments.

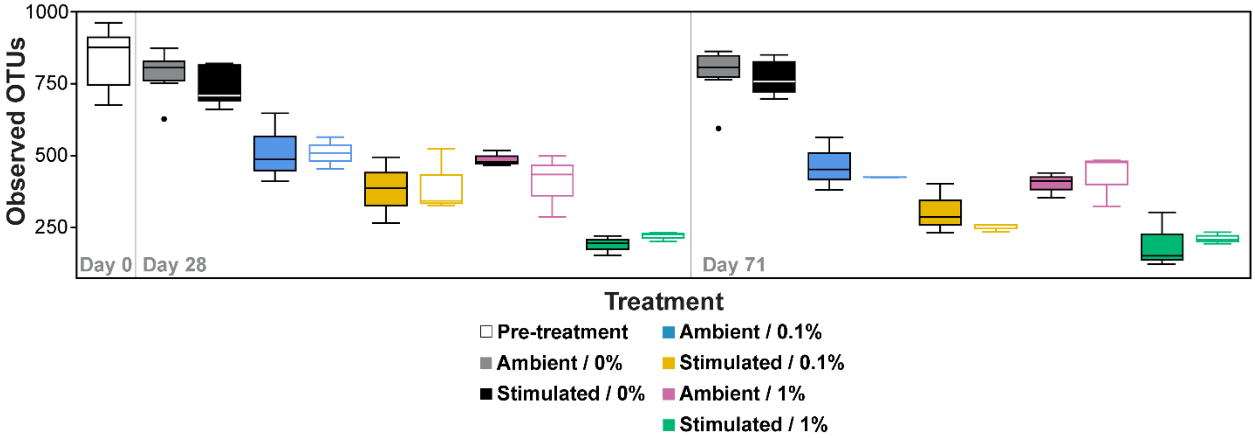

FIG S3: Observed OTUs (i.e., richness) for each treatment group, with the central band representing the median among replicates, the box containing 50% of observations, and whiskers extending to the lowest and highest values excluding outliers (separate points).

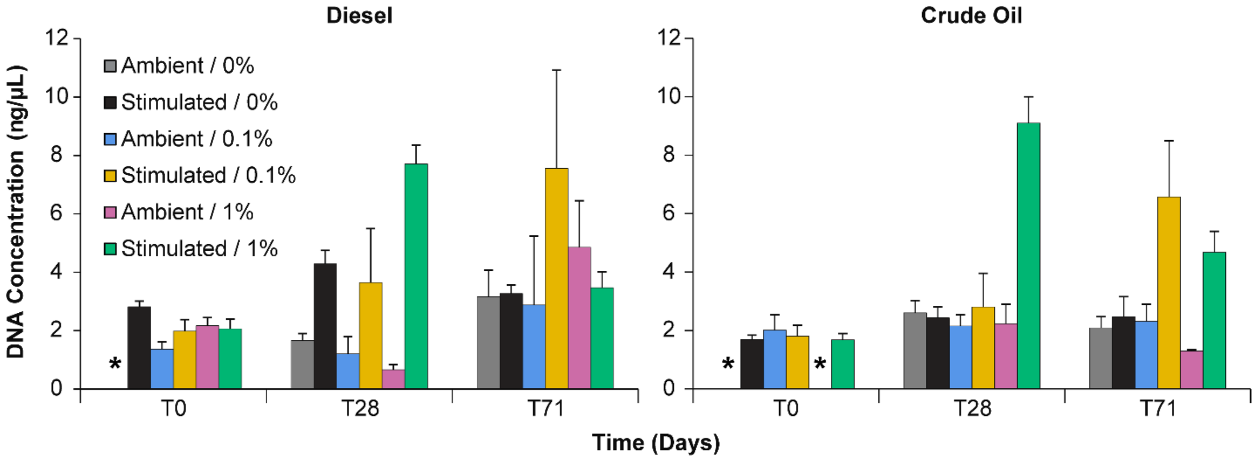

FIG S4: DNA concentrations within treatments and controls at 0, 28, and 71 days of incubation within diesel- and crude oil-amended microcosms. Triplicate microcosms are averaged with standard error bars. Day 0 ambient nutrient treatments marked as * were not analysed since they were not expected to be different from nutrient stimulated treatments.

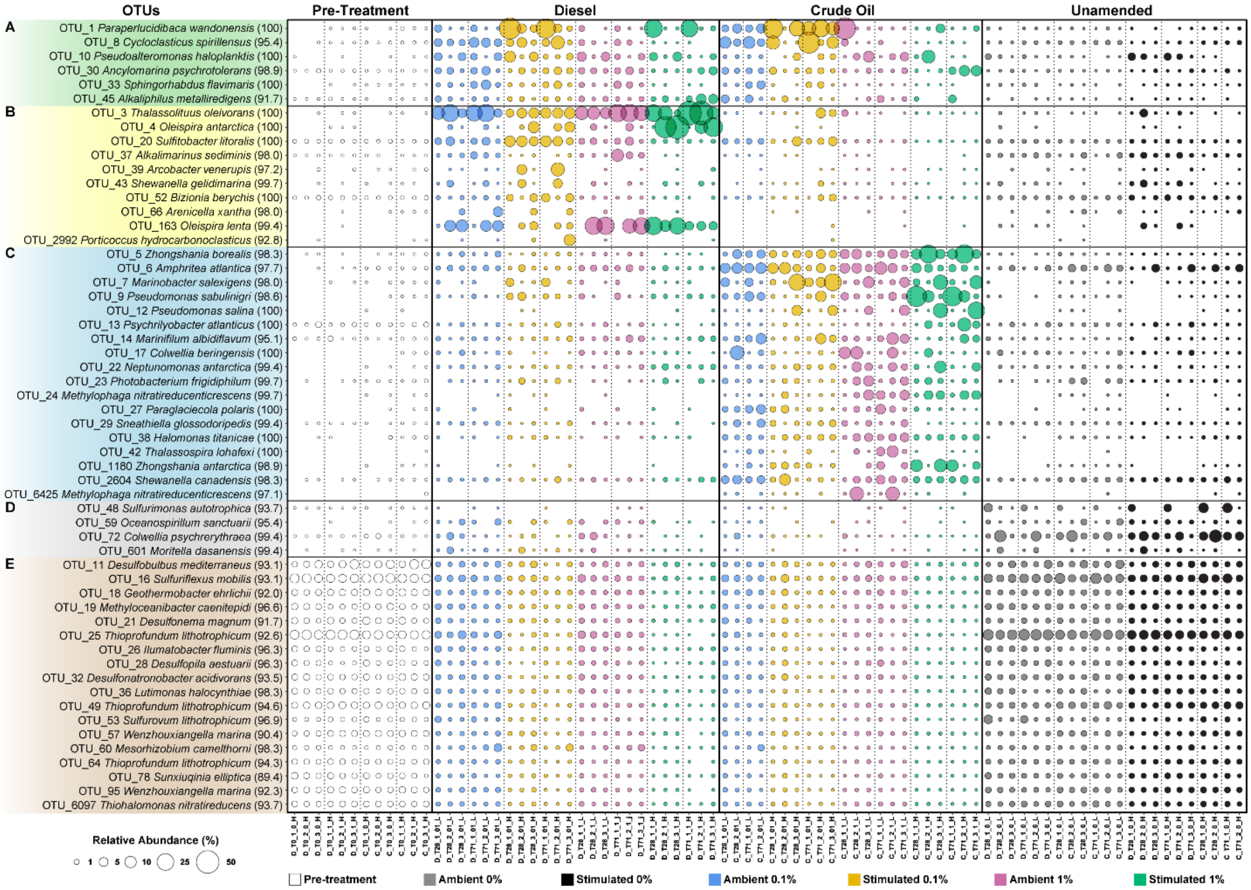

FIG S5: Relative sequence abundance of OTUs with significant associations to diesel or crude oil input after 28 and 71 days of incubation at 4°C (XRF DATASET S4). Bubble size indicates OTU relative abundance (%), with missing bubbles indicating no sequence reads. OTUs are labeled according to closest relatives identified following NCBI BLAST searching with sequence identities (%) shown in parentheses. OTUs are grouped according to significant associations identified by "indicspecies" as those strongly correlated to both diesel and crude oil contamination (A), those correlated to the presence of diesel only (B), crude oil only (C), unamended controls (D), or of the original uncontaminated sediment (E).
